# The adrenal stress response involves distinct dynamics of both cortisol and corticosterone in the axolotl salamander

**DOI:** 10.1038/s41684-026-01692-y

**Published:** 2026-03-02

**Authors:** Anita Dittrich, Sofie Amalie Andersson, Emil A. B. Winkel, Aaron Savage, Steven J. Blair, Kelly E. Dooling, Alexandra C. Wagner, Jessica L. Whited, Catherine J. A. Williams, Henrik Lauridsen

**Affiliations:** 1 Comparative Medicine Lab, Department of Clinical Medicine, Aarhus University, Aarhus, Denmark; 2 Department of Forensic Medicine, Aarhus University, Aarhus, Denmark; 3 Department of Stem Cell and Regenerative Biology, Harvard University, Cambridge, MA USA; 4 Zoophysiology, Department of Biology, Aarhus University, Aarhus, Denmark; 5 Aarhus Institute of Advanced Studies, Aarhus University, Aarhus, Denmark; 6 Department of Animal and Veterinary Science, Aarhus University, Aarhus, Denmark

**Keywords:** Model vertebrates, Animal physiology, Adrenal gland diseases, Steroid hormones

## Abstract

The axolotl is a popular model organism in regenerative biology owing to its ability to regenerate amputated limbs and internal organs. The role of injury-derived signals in initiating the regenerative response is still not well understood, but the potential involvement of the stress response is of interest, as injury and stress are temporally linked. The dominant glucocorticoid response to stress varies among species, with corticosterone generally considered dominant in most amphibians, whereas cortisol predominates in others. Here we characterize the adrenal stress response in the axolotl and describe methods to measure axolotl stress hormones to facilitate their inclusion in future research involving axolotl development and regeneration. We describe an intricate and unexpected axolotl stress response that involves cortisol and corticosterone, each being dominant under different conditions. Corticosterone is preferably activated by the classical hypothalamus–pituitary–interrenal axis pathway, with both arginine vasotocin and adrenocorticotropic hormone promoting its synthesis and release. Under manual stress and direct stimuli with acetylcholine, cortisol is more prominent, suggesting an alternative mechanism involving sympathetic nerve signaling. In response to an amputation injury, both cortisol and corticosterone are increased, with corticosterone being dominant, suggesting an injury-specific response. Finally, when administering glucocorticoids directly and measuring classical physiological effects of glucocorticoid signaling, cortisol is more potent. We propose a hypothesis that axolotls rely on cortisol as their dominant glucocorticoid, functioning in part as an extension of the catecholamine system. By contrast, corticosterone is mainly regulated classically via the hypothalamus–pituitary–interrenal axis.

## Abstract (web summary)

This study examines adrenal stress responses and injury signals in axolotl, showing that corticosterone dominates after amputation, while cortisol, which rises with handling stress, is the primary glucocorticoid in this species.

## Main

The axolotl salamander *Ambystoma mexicanum* (Shaw and Nodder, 1798) is a popular model organism in the fields of aging, development and, most prominently, tissue regeneration, owing to its ability to fully regenerate amputated limbs, as well as damaged or lost tissue in the heart, spinal cord, lung, skin and brain, among others, as extensively reviewed by Yun and Vieira et al.^1,2^. Regenerative research generally requires an initial injury, thereby linking regeneration and the stress response. Furthermore, the role of injury signals involving stress pathways in directly stimulating regenerative processes is not well understood despite being potentially central to the field. Nonetheless, the activation of stress pathways in the axolotl is largely unexplored, and, in fact, the dominant glucocorticoid (GC) is not currently defined.

**Fig. 1:**
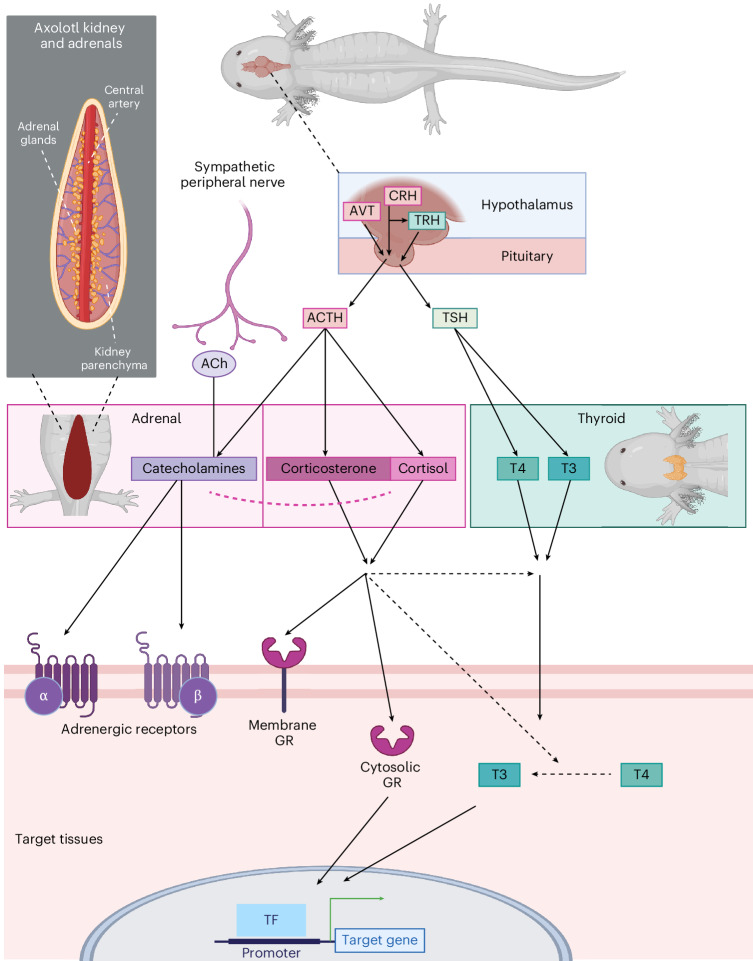
GC and catecholamine stress signaling pathways common to vertebrates. Key elements of the stress pathways involve the adrenal glands situated as disperse interrenal structures in salamanders as well as the hypothalamus and pituitary in the brain. The catecholamine signaling cascade is classically described to be initiated by sympathetic nerves that trigger the production and release of catecholamines (such as adrenaline) from the adrenal glands via ACh signaling. GC signaling (the HPI axis in amphibians) is classically described as being initiated by the release of CRH (in most species) and/or AVT (believed to be more prominent in amphibians), which then triggers the release of ACTH from the pituitary, which in turn stimulates the production and release of the GCs cortisol and/or corticosterone from the adrenal glands. Catecholamines mainly affect target tissues via adrenergic receptors, while GCs bind to extracellular and intracellular GRs, which then act as transcription factors (TFs). There is extensive crosstalk between the HPI and HPT axes, for instance through CRH-induced activation of thyrotropin-releasing hormone (TRH) and GC-driven promotion of the conversion of the inactive thyroid hormone thyroxine (T4) to its active form, triiodothyronine (T3). Figure created with BioRender.com.

Stress-response pathways are highly conserved among vertebrates^3^. Once an animal encounters and senses an environmental stressor, the sympathoadrenal system is activated. Here, the sympathetic nervous system triggers an acute release of catecholamines such as adrenaline from the adrenochromaffin cells in the adrenal tissue via acetylcholine (ACh) neurotransmitter release from preganglionic sympathetic neurons, preparing the animal to initiate a fight or flight response. The adrenal tissue of urodele amphibians (salamanders) is located as clusters of cells along the ventro-medial surface and central vessels of the kidneys^4,5^ (Fig. 1). These structures contain both chromaffin cells and clusters of corticosteroid-producing (steroidogenic) cells; thus, the hypothalamic–pituitary–adrenal (HPA) axis is referred to as the hypothalamic–pituitary–interrenal (HPI) axis in salamanders. This HPI hormonal cascade is activated by an initial release of corticotrophin-releasing hormone (CRH) and/or arginine vasotocin (AVT) from the hypothalamus in the brain^6^. This, in turn, stimulates the pituitary to release adrenocorticotropic hormone (ACTH) into the systemic circulation, which acts on the adrenal steroidogenic cells to synthesize and release GCs into the bloodstream (Fig. 1).

GCs then trigger longer-lasting effects compared with, but complementary to, catecholamines, including adaptations in metabolism, cardiac output, blood pressure, muscle tone and the immune system^7–11^. In amphibians, the HPI axis serves important functions in response to environmental stressors such as water quality, predation and food availability^12–15^. Furthermore, there is a well-established and extensive crosstalk between the HPI and the hypothalamic–pituitary–thyroid (HPT) axes, with the HPT axis controlling and regulating metamorphosis in amphibians^16–26^. Metamorphosis is a developmental process common to amphibians, in which aquatic larvae transform into terrestrial adults, such as the transition from tadpole to frog in anurans. This is especially relevant in axolotls, as they are pedomorphic, meaning they do not normally undergo metamorphosis unless stimulated by exogenous thyroid hormone^27–29^. Importantly, simultaneous treatment with GCs can reduce the dosage of thyroid hormone required for metamorphosis^18,21^, and severe environmental stress can induce spontaneous metamorphosis on its own^30^.

Once GCs enter the bloodstream and diffuse from capillary beds into the tissue, they can bind to membrane-associated cellular receptors^31^ or freely pass through the lipid bilayer of cell membranes in target tissues. Intracellularly, GCs bind to the cytosolic GC receptor (GR) and become translocated to the nucleus where they can exert their primary effects by binding to GC response elements in the DNA and regulate the transcription of target genes^32^. Ultimately, GC signaling is further regulated on many levels, including, but not limited to, negative feedback on CRH, AVT and ACTH, binding of GCs to plasma proteins and a number of interrenal regulatory pathways^33–35^.

The importance of further understanding the GC system in the context of regeneration is highlighted by the fact that tail regeneration is stunted by exposure to corticosterone in axolotls^36^. Furthermore, both tail and skin regeneration are inhibited under the influence of exogenous corticosterone in another species of regenerative salamander (*Desmognathus ochrophaeus*)^37,38^. In zebrafish (*Danio rerio*), another popular model organism of regeneration, chronic stress and elevated cortisol have a negative effect on heart regeneration^39^.

Although the adrenal steroidogenic tissue of most vertebrate species can produce both cortisol and corticosterone, one typically predominates, depending on the species. For instance, cortisol is the primary GC in humans and teleost fish, while corticosterone is most prominent in rodents, birds and reptiles^40^. Corticosterone is also traditionally proposed as the dominant GC in amphibians. In previous studies, different species of anurans as well as salamanders have been shown to display increased release of corticosterone in response to both environmental and manual stress, as well as treatment with ACTH^41–47^. However, there are some cases of especially aquatic amphibians displaying a cortisol-dominant response^48–50^. The eastern hellbender (*Cryptobranchus alleganiensis*) is a pedomorphic salamander like the axolotl^51^. In this species, elevated plasma levels of predominantly cortisol have been reported after both physical restraint and ACTH challenge^50^. A small study using only two axolotls has also demonstrated elevated levels of cortisol in dermal swabs after manual stress in axolotls^52^, leaving it unclear which hormone is most appropriate to measure in the context of stress-pathway activation in axolotls.

In this study, we sought to characterize the adrenal stress response in axolotls, both pharmacologically and via laboratory-relevant biological stress (manual handling and amputation, an example of injury used in regeneration research), to more fully understand how GCs and catecholamines are regulated in response to stress in this species. We also provide methods to measure GCs and catecholamines in axolotls and advocate for the inclusion of these measurements in a broad range of axolotl applications, including developmental, environmental and regenerative studies. We discovered that distinct mechanisms regulate the synthesis and release of cortisol and corticosterone independently, with important implications for future axolotl research.

## Results

### ACTH-challenge test increased both cortisol and corticosterone levels in axolotl plasma, with corticosterone being dominant

**Fig. 2:**
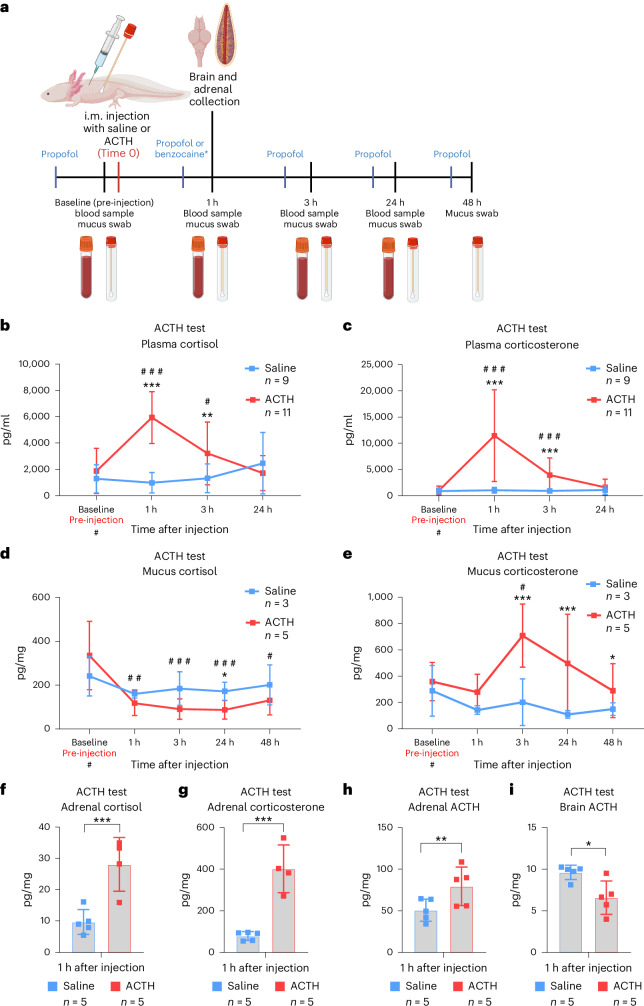
ACTH stress test. **a**, Experimental design. Axolotls were given an i.m. injection of ACTH at 2 mg/kg body mass, and blood samples and mucus swabs were collected at baseline just before the injection and at 1 hpi, 3 hpi, 24 hpi and 48 hpi. Blood samples were centrifuged, and plasma was collected and measured directly, while mucus and tissue samples were homogenized, and GCs were extracted. All non-endpoint data (plasma and mucus) were collected after anesthesia with propofol. Invasive collection of organs was performed under benzocaine anesthesia. GCs and ACTH were assayed by commercial ELISA assays. All groups were of mixed sex, and animals were 1.0–1.5 years old. **b**, Plasma concentration of cortisol increased most prominently 1 h after ACTH administration. Saline *n* = 9, ACTH *n* = 11. **c**, Plasma corticosterone concentration increased most prominently 1 h after ACTH administration. Saline *n* = 9, ACTH *n* = 11. **d**, Mucus sample concentration of cortisol decreased 1 h after ACTH injection. **e**, Corticosterone increased 3 h after ACTH injection. Mucus concentration was normalized to the mass of the sample before extraction. Saline *n* = 3, ACTH *n* = 5. **f**,**g**, Concentrations of cortisol (**f**) and corticosterone (**g**) in adrenal tissue were both higher 1 h after injection with ACTH compared with saline controls. Normalized to mg tissue. Saline *n* = 5, ACTH *n* = 5. **h**,**i**, Concentration of ACTH (combined endogenous or exogenous) per mg tissue sample was higher in adrenal tissue (**h**) and lower in brain tissue (**i**) 1 h after ACTH injection compared with saline controls. Saline *n* = 5, ACTH *n* = 5. Statistical significance was determined by generalized linear mixed modeling with gamma distribution and Šidák (comparison with baseline (#)) or Tukey post-hoc tests (multiple or single pairwise comparison between groups (*)). *P < 0.05, **P < 0.005, ***P < 0.0001. All error bars indicate standard deviation of the data. Squares in bar graphs represent individual replicates. Asterisks indicate statistical significance between saline and ACTH groups and hash marks indicate significance between baseline and other time points within a group. Complete results of statistical analyses are given in Supplementary Table 2. Panel **a** created with BioRender.com.

We performed an ACTH-challenge test equivalent to previous reports in amphibians as well as in clinical practice in humans^50,53^ (Fig. 2a). All animals were first sampled before administration of saline or ACTH to obtain baseline values. All statistical comparisons for this experiment were performed by generalized linear mixed model with gamma distribution (GLMM-γ) and Šidák (comparison within a group across time) or Tukey (comparison between groups) post-hoc tests. After an intramuscular (i.m.) injection with saline, plasma concentrations of both cortisol and corticosterone remained unchanged (Fig. 2b,c). Meanwhile, i.m. injection with ACTH (2 mg/kg body mass) produced an increase in plasma cortisol significantly different from a baseline value of 1,873 ± 1,639 pg/ml at 1 hour after injection (hpi) (5,943 ± 1,883 pg/ml; GLMM-γ and Šidák post-hoc test *z*(4.950), *P* < 0.0001) and 3 hpi (3,209 ± 2,274 pg/ml; GLMM-γ and Šidák post-hoc test *z*(2.578), *P* = 0.0295), before returning to baseline levels by 24 hpi (Fig. 2b). When comparing the saline- and ACTH-injected groups, there was also a significant difference at 1 hpi and 3 hpi (GLMM-γ and Tukey post-hoc tests; *z*(−5.877), *P* < 0.0001, and *z*(−3.017), *P* = 0.0026, respectively), demonstrating that the increase in cortisol was not due to a handling and injection effect but directly caused by the administration of ACTH.

For plasma corticosterone, there was also no significant change after injection in the saline group, while in the ACTH group there was a significant increase from the baseline level (1,009 ± 784 pg/ml) at 1 hpi (11,451 ± 8,342 pg/ml; GLMM-γ and Šidák post-hoc test *z*(9.507), *P* < 0.0001) and 3 hpi (3,932 ± 3,132 pg/ml; GLMM-γ and Šidák post-hoc test *z*(5.637), *P* < 0.0001). There was also a significant difference between the saline- and ACTH-injected groups (GLMM-γ and Tukey post-hoc tests *z*(−6.994), *P* < 0.0001 at 1 hpi; and *z*(−4.251), *P* < 0.0001 at 3 hpi) (Fig. 2c). When comparing the relative fold change from baseline to 1 hpi for both cortisol and corticosterone, we found that corticosterone was significantly more upregulated, suggesting it was the dominant GC after ACTH challenge (GLMM-γ and Tukey post-hoc test; *z*(−5.199), *P* < 0.0001) (Supplementary Fig. 1a).

Mucus swabs were also collected from the lateral body wall to investigate whether a less invasive method of sample collection could be used to detect an increase in GCs. Compared with baseline, no increase in cortisol could be detected in the mucus of either group (Fig. 2d); instead, a statistically significant decrease was observed in the ACTH group at all time points (GLMM-γ and Šidák post-hoc tests; *z*(−3.677), *P* = 0.0009 at 1 hpi; *z*(−4.613), *P* < 0.0001 at 3 hpi; *z*(−4.856), *P* < 0.0001 at 24 hpi; and *z*(−3.394), *P* = 0.0029 at 48 hpi). This was also reflected in a tendency of reduced cortisol in the mucus in the ACTH group compared with saline, which reached statistical significance at 24 hpi (GLMM-γ and Šidák post-hoc test *z*(−1.960), *P* = 0.05). The mechanisms underlying an increase in plasma cortisol alongside a reduction in mucus cortisol are currently unknown. By contrast, corticosterone concentrations in mucus were significantly higher than baseline in the ACTH group at 3 hpi (GLMM-γ and Šidák post-hoc test; *z*(2.573), *P* = 0.0398) before returning to baseline over the course of 48 hpi (Fig. 2e). When comparing the saline and ACTH groups, mucus corticosterone was significantly higher in the ACTH group (GLMM-γ and Tukey post-hoc tests; *z*(3.920), *P* < 0.0001 at 3 hpi; *z*(4.247), *P* < 0.0001 at 24 hpi; and *z*(2.019), *P* = 0.0434 at 48 hpi).

These results show that, in response to exogenous ACTH challenge, axolotls released both cortisol and corticosterone into systemic circulation, with peak plasma concentrations at 1 hpi and a more pronounced upregulation of corticosterone (Supplementary Fig. 1a). Only corticosterone was increased in mucus samples, with the highest concentration measured in the samples collected at 3 hpi. It is currently unknown whether mucus GCs are regulated by diffusion from the plasma or lymph or by some other mechanism.

### ACTH administration increased GC concentrations within the adrenal tissue

While adrenal tissue samples from the saline group contained 9.66 ± 3.56 pg/mg cortisol and 78.72 ± 18.64 pg/mg corticosterone at 1 hpi, samples from the ACTH-injected group contained significantly higher levels of cortisol (28.08 ± 7.45 pg/mg) and corticosterone (401.84 ± 99.55 pg/ml) (GLMM-γ and Tukey post-hoc tests *z*(−5.389), *P* < 0.0001 for cortisol; and *z*(−8.130), *P* < 0.0001 for corticosterone) (Fig. 2f,g). When comparing fold changes in GC concentrations with the saline group, the increase in corticosterone was greater than that of cortisol (GLMM-γ and Tukey post-hoc test *z*(−4.317), *P* < 0.0001) (Supplementary Fig. 1b). These results show that ACTH not only stimulated the release of both GCs into circulation but also their synthesis in adrenal tissue, with corticosterone being dominant.

### Exogenous ACTH downregulated endogenous ACTH production

Adrenal tissue at 1 hpi had a higher concentration of ACTH after injection with exogenous ACTH compared with saline controls (Fig. 2h) with 50.78 ± 11.92 pg/mg tissue in the saline group and 79.48 ± 20.67 pg/mg tissue in the ACTH group (GLMM-γ and Tukey post-hoc test *z*(−3.080), *P* = 0.0021). Brain samples collected 1 h after injection of exogenous ACTH, meanwhile, had a significantly lower ACTH level compared with the brains of saline-injected animals (Fig. 2g) with 9.61 ± 0.76 pg/mg tissue in the saline group and 6.57 ± 1.79 pg/mg tissue in the ACTH group (GLMM-γ and Tukey post-hoc test; *z*(2.610), *P* = 0.0091). These results demonstrated that, while exogenous ACTH injection resulted in higher concentrations in the adrenals (which may have been of endogenous or exogenous origin), higher levels of systemic ACTH may induce a negative feedback mechanism to limit further production of ACTH in the brain.

### Axolotls produced endogenous ACTH when administered AVT

**Fig. 3:**
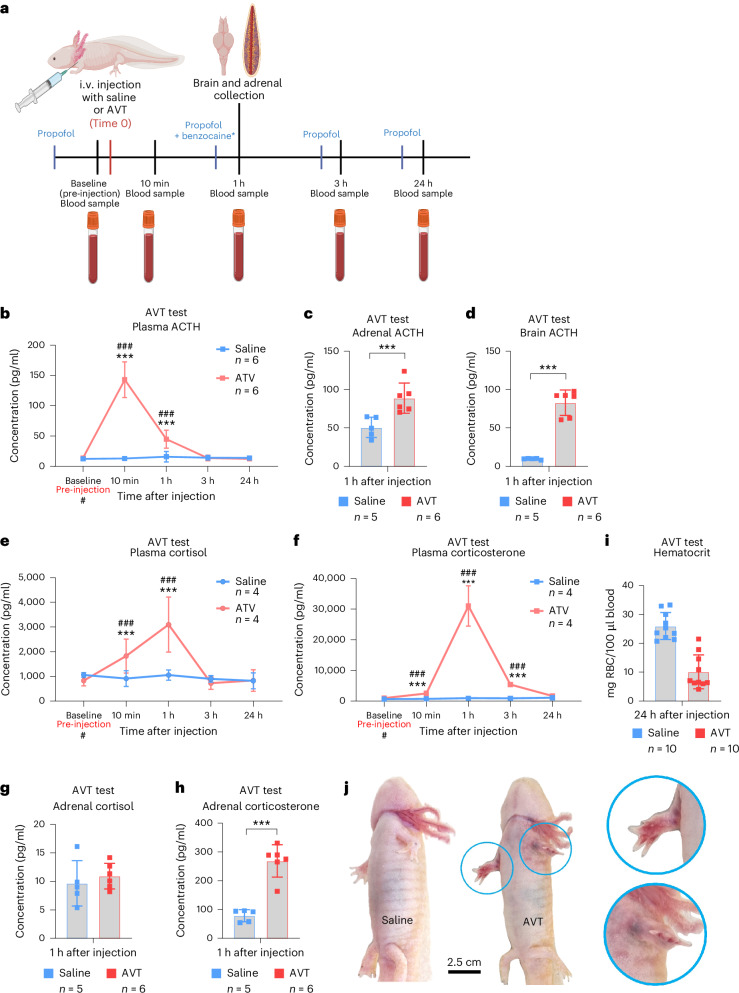
AVT stress test. **a**, Experimental design. Axolotls were given an i.v. injection of AVT at 1 µg/g body mass. Blood samples were collected at baseline before injection at 10 mpi, 1 hpi, 3 hpi and 24 hpi. Tissue samples were collected at 1 hpi. Blood samples were centrifuged, and plasma was collected and measured directly, while mucus and tissue samples were homogenized, and GCs were extracted. All non-endpoint data (plasma and mucus) were collected after anesthesia with propofol. Invasive collection of organs was performed under benzocaine anesthesia. GCs and ACTH were assayed by commercial ELISA assays. All groups were of mixed sex, and animals were 1.0–1.5 years old. **b**, Plasma ACTH concentration increased most prominently 10 min after AVT challenge and remained elevated at 1 hpi, but to a lesser extent. Saline *n* = 6, ACTH *n* = 6. **c**, ACTH concentration was higher in adrenal tissue 1 h after AVT injection compared with saline controls. Normalized to tissue mass. Saline *n* = 5, ACTH *n* = 6. **d**, ACTH concentration was higher in brain tissue 1 h after AVT injection compared with saline controls. Normalized to tissue mass. Saline *n* = 5, ACTH *n* = 6. **e**, Plasma cortisol concentration had increased by 10 min after AVT injection and further increased after 1 h. Saline *n* = 4, ACTH *n* = 4. **f**, Plasma corticosterone concentration increased most prominently 1 h after AVT injection. Saline *n* = 4, ACTH *n* = 4. **g**, Cortisol concentration in adrenal tissue was unaffected 1 h after AVT injection. Normalized to tissue mass. Saline *n* = 5, ACTH *n* = 6. **h**, Corticosterone concentration was higher in adrenal tissue 1 h after AVT injection compared with saline controls. Normalized to tissue mass. Saline *n* = 5, ACTH *n* = 6. **i**, Hematocrit, measured as the red blood cell (RCB) mass-to-volume ratio 24 h after AVT injection, was decreased in the AVT group compared with the saline control. Saline *n* = 10, ACTH *n* = 10. **j**, We observed subcutaneous bleeding effects (blue circles) of AVT injection that were not seen in saline-injected animals. Statistical significance was determined by generalized linear mixed modeling with gamma distribution and Šidák (comparison with baseline (#)) or Tukey post-hoc tests (multiple or single pairwise comparison between groups (*)). *P < 0.05, **P < 0.005, ***P < 0.0001. All error bars indicate standard deviation of the data. Squares in bar graphs represent individual replicates. Asterisks indicate statistical significance between saline and ACTH groups and hash marks indicate significance between baseline and other time points within a group. Complete results of statistical analyses are given in Supplementary Table 2. Panel **a** created with BioRender.com.

After establishing that axolotls can produce both GCs, albeit at different concentrations in response to ACTH, we next investigated whether axolotls produce endogenous ACTH and whether upstream stimuli elicit similar effects on cortisol and corticosterone, respectively. While CRH is the primary regulator of ACTH release in mammals, AVT is thought to have this role in amphibians^4^, with CRH being possibly more relevant to the HPT axis^16,17^. We therefore injected axolotls with saline or AVT (1 µg/g body mass) via intravenous (i.v.) injection in the jugular vein (Supplementary Fig. 2) and collected blood and tissue samples (Fig. 3a). All animals were first sampled before administration of saline or AVT to obtain baseline values. All statistical comparisons for this experiment were performed by GLMM-γ and Šidák (comparison within a group across time) or Tukey (comparison between groups) post-hoc tests.

While the saline-injected group showed no significant change in plasma ACTH levels from baseline, ACTH levels increased significantly in response to AVT challenge. Levels increased from 13.96 ± 1.76 pg/ml at baseline to 143.03 ± 26.83 pg/ml at 10 minutes post-injection (mpi) (GLMM-γ and Šidák post-hoc test; *z*(17.80), *P* < 0.0001), and to 44.86 ± 13.55 pg/ml at 1 hpi (GLMM-γ and Šidák post-hoc test *z*(8.907), *P* < 0.0001), before returning to baseline levels by 3 hpi (Fig. 3b). When comparing the saline- and AVT-injected animals, we also found a significant difference (GLMM-γ and Tukey post-hoc tests; *z*(18.321), *P* < 0.0001 at 10 mpi; and *z*(7.863), *P* < 0.0001 at 1 hpi).

Injection with AVT also significantly increased ACTH levels in both adrenal and brain tissue at 1 hpi, with ACTH levels found to be 88.82 ± 18.03 pg/mg in adrenal tissue (Fig. 3c) and 82.76 ± 15.09 pg/mg in brain tissue (Fig. 3d). These ACTH tissue concentrations were both significantly different from the saline group, which contained 50.78 pg/ml ACTH in adrenal tissue and 9.61 ± 0.76 pg/ml in brain tissue (GLMM-γ and Tukey post-hoc tests; *z*(−4.922), *P* < 0.0001 for adrenal tissue; and *z*(−17.794), *P* < 0.0001 for brain tissue).

### After AVT injection, axolotls produced both cortisol and corticosterone, with corticosterone being dominant

Downstream, AVT injection had similar effects to ACTH injection on plasma cortisol and corticosterone. Again, the concentrations of both GCs increased (Figs. 3e,f), but corticosterone levels increased more in terms of fold change from baseline (Supplementary Fig. 1c).

In the AVT group, cortisol increased significantly from a baseline plasma level of 833 ± 185 pg/ml to a level of 1,827 ± 596 pg/ml at 10 mpi (GLMM-γ and Šidák post-hoc test; *z*(4.164), *P* < 0.0001) and to a level of 3,097 ± 966 pg/ml at 1 hpi (GLMM-γ and Šidák post-hoc test; *z*(6.951), *P* < 0.0001). When comparing the saline and AVT group, there was a significant difference in plasma cortisol levels between the two groups at 10 mpi and 1 hpi (GLMM-γ and Tukey post-hoc tests *z*(3.676), *P* = 0.0002 and *z*(5.676), *P* < 0.0001) (Fig. 3e).

Corticosterone increased significantly from a baseline plasma level of 1,008 ± 176 pg/ml to a level of 2,505 ± 643 pg/ml at 10 mpi, 31,057 ± 5,696 pg/ml at 1 hpi and 5,409 ± 245 pg/ml at 3 hpi (GLMM-γ and Šidák post-hoc tests; *z*(4.807), *P* < 0.0001 at 10 mpi; *z*(18.138), *P* < 0.0001) at 1 hpi; *z*(8.908), *P* < 0.0001 at 3 hpi). When comparing the saline- and AVT-injected groups, there were also significant differences in plasma corticosterone at 10 mpi, 1 hpi and 3 hpi (GLMM-γ and Tukey post-hoc tests; *z*(6.273), *P* < 0.0001; *z*(18.229), *P* < 0.0001 and *z*(9.225), *P* < 0.0001, respectively) (Fig. 3f). Furthermore, there was a significant difference in the fold change from baseline for cortisol versus corticosterone at the 1 hpi peak, indicating that the increase in corticosterone was dominant at this time (GLMM-γ and Tukey post-hoc test; *z*(−10.960), *P* < 0.0001) (Supplementary Fig. 1c).

Adrenal tissue did not show an increased concentration of cortisol at 1 hpi after AVT injection (9.66 ± 3.56 pg/mg in saline group versus 10.92 ± 2.05 pg/mg in AVT group) (Fig. 3g), but showed a significant increased concentration of corticosterone (78.72 ± 18.63 pg/mg in the saline group versus 269 ± 51.58 pg/mg in the AVT group) (Fig. 3h) (GLMM-γ and Tukey post-hoc test; *z*(−7.999), *P* < 0.0001). Given that the plasma ACTH peak was already observed 10 mpi after AVT administration, while the highest concentrations of GCs were measured at 1 hpi, it is possible that the amount of cortisol produced in response to AVT injection had already been released into the bloodstream by 1 hpi, leading to there being no difference in the adrenal tissue. Alternatively, the rate of synthesis versus release may differ between cortisol and corticosterone.

Overall, the results from the AVT test demonstrated that an i.v. injection of AVT activated the HPI axis via production and release of ACTH from the pituitary and subsequently led to an increase of ACTH in the adrenal tissue prompting the synthesis and release of both GCs, but most prominently corticosterone. We also noted some side effects after AVT injection, including a decrease in hematocrit still present 24 h after injection (Fig. 3i) as well as subcutaneous bleeding and bruising in the extremities that persisted for 2–3 days (Fig. 3j).

### Manual stress also increased plasma ACTH levels

After concluding that the axolotl HPI axis could induce rises in concentration of both cortisol and corticosterone, with corticosterone being dominant, we also wanted to determine the dynamics of the response to manual stress. The dosages of AVT and ACTH that we previously administered probably mimic a severe stress response and/or could have been administered at supraphysiological levels. Therefore, a manual test would be more relevant to the animal’s response to mild or moderate stressors typically experienced during handling and animal experiments.

**Fig. 4:**
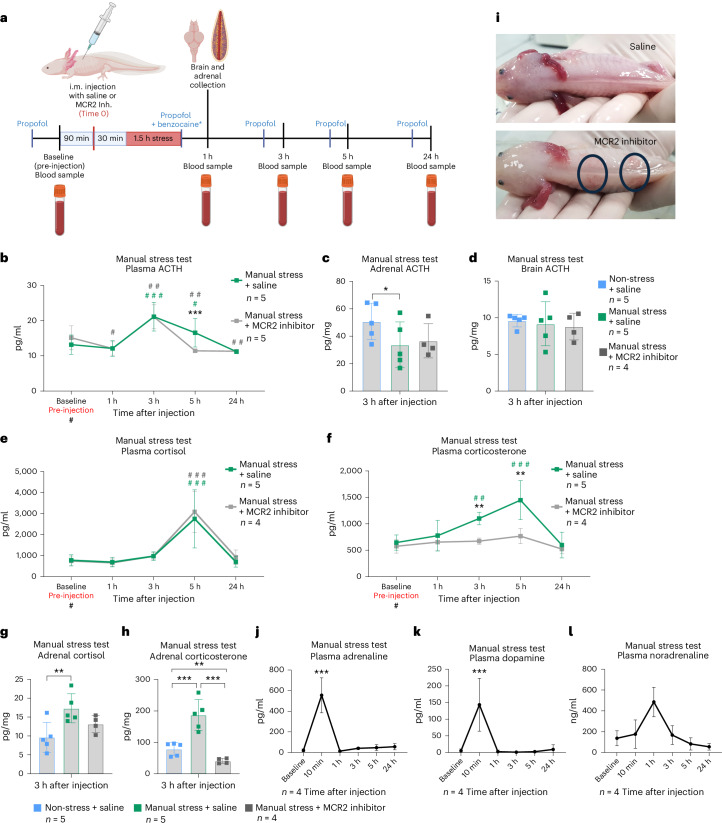
Manual stress test. **a**, Experimental design. Axolotls were exposed to manual stress simulating transportation and handling by lowering the water level in their housing crates, intermittently shaking them for 1.5 h, and briefly lifting them out of the water. Blood samples were collected at baseline before injection and at 1 hpi, 3 hpi, 5 hpi and 24 hpi. Tissue samples were collected at 1 hpi. Blood samples were centrifuged, and plasma was collected and measured directly, while mucus and tissue samples were homogenized, and GCs were extracted. All non-endpoint data (plasma and mucus) were collected after anesthesia with propofol. Invasive collection of organs was done under benzocaine anesthesia. GCs and ACTH were assayed by commercial ELISA assays. All groups were of mixed sex, and animals were 1.0–1.5 years old. **b**, Plasma ACTH concentration increased 3 h after manual stressor with administration of both saline and melanocortin 2 receptor (MCR2) antagonist. Manual stress with saline group *n* = 5, manual stress with inhibitor group *n* = 5. **c**, ACTH concentration was lower in the manual stress with saline group (but not in the manual stress with inhibitor group) in adrenal tissue 3 h after stressor compared with the non-stress saline control. Normalized to tissue mass. Non-stress saline *n* = 5, manual stress with saline group *n* = 5, manual stress with inhibitor group *n* = 4. **d**, ACTH concentrations were unaffected in brain tissue 3 h after stressor. Normalized to tissue mass. Non-stress saline *n* = 5, manual stress with saline group *n* = 5, manual stress with inhibitor group *n* = 4. **e**, Plasma cortisol concentration increased 5 h after manual stress in both groups (with saline or inhibitor). Manual stress with saline group *n* = 5, manual stress with inhibitor group *n* = 4. **f**, Plasma corticosterone concentration increased from 3 h and most prominently at 5 h after manual stress in the manual stress with saline group, but remained unaffected in the inhibitor group. Manual stress with saline group *n* = 5, manual stress with inhibitor group *n* = 4. **g**, Tissue concentration of cortisol was higher in the manual stress with saline group (but not in the manual stress with inhibitor group) compared with the non-stress saline control in adrenals 3 h after manual stress. Non-stress saline *n* = 5, manual stress with saline group *n* = 5, manual stress with inhibitor group *n* = 4. **h**, Tissue concentration of corticosterone was higher in the manual stress with saline group compared to the non-stress saline control, while lower compared with the non-stress saline control in the inhibitor group in adrenal tissue 3 h after manual stress. Non-stress saline *n* = 5, manual stress with saline group *n* = 5, manual stress with inhibitor group *n* = 4. **i**, Observed local vasodilatory effects at the injection sites 1 h after injection of saline and MCR2 antagonist, highlighted in blue circles. **j**–**l**, Plasma concentrations of catecholamines adrenaline (**j**), dopamine (**k**) and noradrenaline (**l**) after manual stress. Adrenaline and dopamine both increased 10 min after stressor and quickly returned to normal, whereas noradrenaline showed a tendency to increase (but not statistically significant) 1 h after stressor. *n* = 4. Statistical significance was determined by generalized linear mixed modeling with gamma distribution and Šidák (comparison with baseline (#)) or Tukey post-hoc tests (multiple or single pairwise comparison between groups (*)). *P < 0.05, **P < 0.005, ***P < 0.0001. All error bars indicate standard deviation of the data. Squares in bar graphs represent individual replicates. Asterisks indicate statistical significance between saline and ACTH groups, while hash marks indicate significance between baseline and other time points within a group. Complete results of statistical analyses are given in Supplementary Table 2. Panel **a** created with BioRender.com.

We exposed unanesthetized axolotls to 1.5 h of manual stress by lowering the water level of their housing crates and shaking the crate for 2.5 min every 5 min before picking the animals up and out of the water ten times in quick succession at the end of the stressor period (simulating transportation and handling relevant to experimental settings). Blood samples and tissue samples were collected at relevant time points (blood samples were also obtained before stress from all animals to obtain baseline values) (Fig. 4a). To elucidate the role of ACTH during this manual stress, we also included a group that was administered a melanocortin receptor type 2 (MCR2) antagonist (with non-inhibited animals given an equivalent volume of saline). MCR2 is the ACTH receptor expressed by GC-producing cells in the adrenals, thus mediating HPI-axis-driven synthesis and release of GCs. All statistical comparisons for this experiment were performed by GLMM-γ and Šidák (comparison within a group across time) or Tukey (comparison between groups) post-hoc tests.

After manual stress, we measured a significantly increased level of ACTH in plasma compared with baseline at 3 hours post manual stress (hpms) in both groups (with administration of saline or inhibitor) (Fig. 4b). In the manual stress group with saline injection, values increased from baseline (13.19 ± 2.37 pg/ml) to 3 hpms (21.13 ± 4.98 pg/ml) (GLMM-γ and Šidák post-hoc test; *z*(5.567), *P* < 0.0001) and remained elevated at 5 hpms (GLMM-γ and Šidák post-hoc test; *z*(2.683), *P* = 0.0289). In the manual stress with inhibitor group, ACTH levels increased from baseline levels of 15.14 ± 3.12 pg/ml to 21.09 ± 4.61 pg/ml at 3 hpms (GLMM-γ and Šidák post-hoc test; *z*(3.942), *P* = 0.0003); however, ACTH levels were no longer increased at 5 hpms. This difference was also observed when comparing the two groups, where a significant difference was confirmed at 5 hpms (GLMM-γ and Tukey post-hoc test; *z*(−3.935), *P* < 0.0001), which could suggest that MCR2 inhibition works in a negative feedback mechanism to impact the upstream production of ACTH at this time.

We also detected a modest decrease in ACTH levels in adrenal tissue at 3 hpms in the manual stress + saline group compared with the non-stressed saline group (GLMM-γ and Tukey post-hoc test; *z*(2.577), *P* = 0.0269). While a similar tendency was seen in the inhibitor group, this was not statistically different from non-stressed saline or the manual stress with saline group (Fig. 4c). No difference in ACTH levels was seen between the groups in brain tissue at 3 hpms (Fig. 4d). These findings indicate that, while manual stress could produce a measurable increase in plasma ACTH (although less dramatic compared with the response to AVT), a similar effect was not observed in the tissues, at least not at the time of tissue collection (3 hpms).

### Unlike activation via AVT or ACTH, manual stress induced a cortisol-dominated response

Manual stress with saline injection led to a significant increase in both plasma cortisol and corticosterone from baseline levels, with an observed peak at 5 hpms (Fig. 4e,f). Unlike the findings with AVT or ACTH challenge, cortisol was dominant in absolute levels and fold change at the 5 hpms peak (GLMM-γ and Tukey post-hoc tests; *z*(6.065), *P* < 0.0001 (absolute levels) and *P* = 0.0130 (fold change)) (Supplementary Fig. 1d). Cortisol plasma concentrations went from 781 ± 235 pg/ml at baseline to 2,750 ± 847 pg/ml at 5 hpms in the manual stress with saline group (GLMM-γ and Šidák post-hoc test; *z*(7.649), *P* < 0.0001) (Fig. 4e). Corticosterone increased in the manual stress with saline group from 645 ± 127 pg/ml at baseline to 1,447 pg/ml at 5 hpms (GLMM-γ and Šidák post-hoc test; *z*(4.902), *P* < 0.0001) (Fig. 4f). In addition, a significant increase from baseline was also present earlier at 3 hpms for corticosterone (GLMM-γ and Šidák post-hoc test; *z*(3.237), *P* = 0.0048), but not cortisol (Fig. 4e,f).

In adrenal tissue, the concentrations of cortisol and corticosterone were also both increased when comparing tissue collected from the non-stressed saline-injected animals and the manual stress with saline group (Fig. 4g), with cortisol concentrations in non-stressed saline-injected animals at 9.66 ± 3.56 pg/mg and in manually stressed with saline animals at 17.31 ± 3.45 pg/mg (GLMM-γ and Tukey post-hoc test; *z*(−4.001), *P* = 0.0002). Adrenal corticosterone concentration was 78.72 ± 18.64 pg/mg in the non-stressed saline group and 187 ± 43.83 pg/mg in the manual stress with saline group (GLMM-γ and Tukey post-hoc test *z*(−5.737), *P* < 0.0001). When comparing fold changes relative to baseline, the increase in corticosterone was greater than that of cortisol (GLMM-γ and Tukey post-hoc test; *z*(2.187), *P* < 0.0001) (Supplementary Fig. 1e). Overall, these results showed that manually induced stress resulted in a cortisol-dominant release of GCs into the circulation, although the synthesis of both GCs was increased in the adrenals.

### Antagonism of MCR2 inhibited the production and release of corticosterone, but not cortisol

In the manual stress with inhibitor group, plasma cortisol increased from 750 ± 205 pg/ml at baseline to 3,085 ± 847 pg/ml at 5 hpms (GLMM-γ and Šidák post-hoc test; *z*(7.649), *P* < 0.0001), which was not significantly different from the manual stress with saline group (Fig. 4e). For corticosterone, there was no significant difference between baseline and 5 hpms in the manual stress with inhibitor group, unlike the manual stress with saline group; thus, MCR2 inhibition led to a significant difference between the two groups at both 3 hpms (GLMM-γ and Tukey post-hoc test; *z*(2.823), *P* = 0.0048) and 5 hpms (GLMM-γ and Tukey post-hoc test; *z*(3.631), *P* = 0.0003) (Fig. 4f and Supplementary Fig. 1d).

Similarly, the adrenal tissue did not show a significant difference in cortisol concentration when comparing the manual stress with saline and manual stress with inhibitor groups (Fig. 4g and Supplementary Fig. 1e), while a significant difference was found in adrenal tissue corticosterone concentrations between the same two groups (GLMM-γ and Tukey post-hoc test; *z*(9.420), *P* < 0.0001) (Fig. 4h and Supplementary Fig. 1e).

We observed a vasodilatory effect locally at the injection site in the animals administered the MCR2 antagonist that was not seen in the saline-injected animals (Fig. 4i). Overall, these results show that corticosterone increase after manual stress was more dependent on the classical activation pathway via the HPI axis and ACTH binding to the adrenal MCR2 receptor, while cortisol was seemingly regulated at least in part via an ACTH-independent mechanism.

### Manual stress induced a rapid release of adrenaline and dopamine followed by a delayed release of noradrenaline

We measured the release of catecholamines in the plasma after the manual stress test to map the possible timing of any crosstalk between catecholamines and GCs. We found that adrenaline rapidly increased in the plasma from 0.0356 ± 0.0193 ng/ml at baseline to 0.5551 ± 0.1468 ng/ml 10 minutes after manual stress (mpms) (GLMM-γ and Šidák post-hoc test; *z*(6.295), *P* < 0.0001) (Fig. 4j). By 1 hpms, levels had already normalized and remained low throughout the measured time points at 3 hpms, 5 hpms and 24 hpms. We saw a similar dynamic in plasma dopamine levels (Fig. 4k), which increased significantly from 0.0052 ± 0.0049 ng/ml at baseline to 0.1435 ± 0.0687 ng/ml by 10 mpms (GLMM-γ and Šidák post-hoc test; *z*(6.438), *P* < 000.1). Noradrenaline showed a tendency of increased plasma concentration at a delayed time point compared with adrenaline and dopamine (Fig. 4l), although this increase was not significant.

These results demonstrated that the acute effects of manual stress could be detected by the presence of catecholamines in axolotl plasma, within a time frame that would allow the catecholamine and GC systems to interact. Adrenaline and dopamine increased most rapidly with a significant increase already observed at 10 mpms; levels returned to baseline very quickly. Noradrenaline showed a tendency of increasing at 1 hpms.

### Amputation injury increased both cortisol and corticosterone levels

**Fig. 5:**
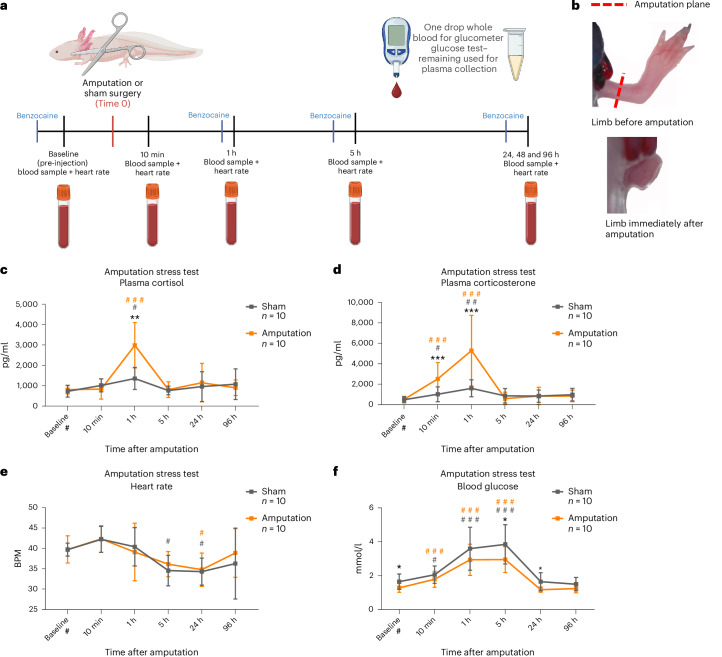
Amputation injury stress test. **a**, Experimental design. Sham or amputation surgery was performed in axolotls and blood samples collected at baseline before surgery and at 10 min post amputation (mpa)/sham, 1 hpa/sham, 5 hpa, 24 hpa, 48 hpa and 96 hpa. Whole blood was immediately used for glucose measurements with a glucometer device, and the remaining blood samples were centrifuged and plasma was collected and measured directly. GCs were assayed by commercial ELISA assays. All groups were of mixed sex, and animals were 1.0–1.5 years old. **b**, Representative images of the limb before and after amputation. The dotted red line marks the amputation plane. **c**, Plasma cortisol concentrations increased 1 h after amputation injury compared with sham controls. Sham *n* = 10, amputation *n* = 10. **d**, Plasma corticosterone concentrations increased from 10 min and most prominently 1 h after amputation injury compared with sham controls. Sham *n* = 10, amputation *n* = 10. **e**, Heart rate measured by echocardiography was decreased 5 h and 24 h after both sham and amputation injury. Sham *n* = 10, amputation *n* = 10. **f**, Blood glucose measured by glucometer increased after both sham and amputation injury. Sham *n* = 10, amputation *n* = 10. Statistical significance was determined by generalized linear mixed modeling with gamma distribution and Šidák (comparison with baseline (#)) or Tukey post-hoc tests (multiple or single pairwise comparison between groups (*)). *P < 0.05, **P < 0.005 and ***P < 0.0001. All error bars indicate standard deviation of the data. Squares in bar graphs represent individual replicates. Asterisks indicate statistical significance between saline and ACTH groups and hash marks indicate significance between baseline and other time points within a group. Complete results of statistical analyses are given in Supplementary Table 2. Panel **a** created with BioRender.com.

Considering the complex stress responses observed under differing conditions and the possible role of GC signaling in injury and regeneration, we also performed a stress test by performing an amputation injury or sham injury (Fig. 5a). Amputating the axolotl forelimb mid-humerus under anesthesia is a commonly used injury model to study limb regeneration (Fig. 5b). We included a later time point of 96 h (4 days post injury) to include a time during blastema formation, which is an early critical step in limb regeneration^54^. The aim was to determine whether GCs remained elevated and potentially played an important role at this time point. All statistical comparisons for this experiment were performed by GLMM-γ and Šidák (comparison within a group across time) or Tukey (comparison between groups) post-hoc tests.

We found that plasma cortisol concentration was significantly upregulated at 1 hour post amputation (hpa)/sham compared with baseline (increasing from 807 ± 191 pg/ml to 2,981 ± 1,050 pg/ml) (GLMM-γ and Šidák post-hoc test; *z*(5.278), *P* < 0.0001) in the amputation group. Plasma cortisol was also increased in the sham group at 1 h post-surgery (increasing to 1,357 ± 510 pg/ml) (GLMM-γ and Šidák post-hoc test; *z*(2.599), *P* = 0.0458) (Fig. 5c). Importantly, when comparing the sham and amputation group, the peak in cortisol concentration was significantly higher after amputation (GLMM-γ and Tukey post-hoc test; *z*(3.022), *P* = 0.0025) (Fig. 5c). For corticosterone, a significant increase from baseline levels (518 ± 260 pg/ml) occurred earlier in the amputation group, with a significant increase already observed at 10 min (increasing to 2,516 ± 1,502 pg/ml) (GLMM-γ and Šidák post-hoc test; *z*(2.834), *P* < 0.0001). This increase was followed by a peak at 1 hpa (5,279 ± 3,278 pg/ml; GLMM-γ and Šidák post-hoc test; *z*(4.760), *P* < 0.0001). A similar time course was observed in the sham group, but to a lesser extent (Fig. 5d). When comparing the sham and amputation groups, a significant difference in plasma corticosterone was observed at 10 min (GLMM-γ and Tukey post-hoc test; *z*(3.664), *P* = 0.0002) and at 1 hpa (GLMM-γ and Tukey post-hoc test; *z*(4.873), *P* < 0.0001). Furthermore, the fold change from baseline in the amputation group was significantly higher for plasma corticosterone than for plasma cortisol (16-fold versus 3.8-fold) at the 1 h observed peak (GLMM-γ and Tukey post-hoc test *z*(−5.130), *P* < 0.0001) (Supplementary Fig. 1f). Interestingly, the plasma increase in corticosterone was more rapid after both manual stress (Fig. 4) and amputation injury (Fig. 5), compared with the increase observed after upstream activation of the HPI axis, with currently unknown downstream implications.

Overall, these results demonstrated that injury could produce a response involving both cortisol and corticosterone, with corticosterone increasing earlier and to a greater extent. This response was different from manual stress in the absence of injury. However, the fold change from baseline between plasma cortisol and corticosterone levels was not as markedly different as after the ACTH or AVT tests (Supplementary Fig. 1a,c and versus Supplementary Fig. 1f). Importantly, the elevation in both GCs did not last beyond 5 h and was thus no longer different from baseline during the later phase of regeneration at 96 hpa.

### Sham and amputation injury had similar effects on heart rate and blood glucose levels

Heart rate in both sham and amputation-injured animals decreased from baseline (39.68 ± 1.5 beats per minute (BPM) (sham) and 39.72 ± 3.14 BPM (amputation)) at 24 hpa (34.53 ± 3.55 BPM for sham (GLMM-γ and Šidák post-hoc test; *z*(−2.970), *P* = 0.0148) and 36.11 ± 2.93 BPM for amputation (GLMM-γ and Šidák post-hoc test; *z*(−2.709), *P* = 0.0.033). However, there was no statistical difference between the groups (Fig. 5e).

We also measured blood glucose as elevated plasma glucose is a known physiological effect of elevated GCs given that they activate enzymes involved in gluconeogenesis in the liver while inhibiting glucose uptake of peripheral tissues like skeletal muscle^55^. We found that blood glucose levels increased compared with baseline (1.64 ± 0.43 mmol/l in sham group and 1.28 ± 0.24 mmol/l in amputation group) in both groups at 10 min (2.06 ± 0.48 mmol/l for sham (GLMM-γ and Šidák post-hoc test; *z*(3.247), *P* = 0.0058) and 1.78 ± 0.45 mmol/l for amputation (GLMM-γ and Šidák post-hoc test; *z*(4.265), *P* < 0.0001)). This was also the case at 1 hpa (3.59 ± 1.20 mmol/l for sham (GLMM-γ and Šidák post-hoc test; *z*(10.168), *P* < 0.0001) and 2.93 ± 0.87 mmol/l for amputation (GLMM-γ and Šidák post-hoc test; *z*(10.780), *P* < 0.0001)) and at 5 hpa (3.85 ± 1.10 mmol/l for sham (GLMM-γ and Šidák post-hoc test; *z*(11.335), *P* < 0.0001) and 2.95 ± 0.73 mmol/l for amputation (GLMM-γ and Šidák post-hoc test; *z*(11.036), *P* < 0.0001)) (Fig. 5f). When comparing the two groups, a significant difference was observed, with the amputation group having a lower blood glucose at 5 hpa (GLMM-γ and Tukey post-hoc test *z*(−2.13), *P* = 0.0269) and 24 hpa (GLMM-γ and Tukey post-hoc test *z*(−2.623), *P* = 0.0087). Increased GCs would be expected to elevate both heart rate and blood glucose, and it is unknown at this time why an increase in heart rate was not seen in association with increased plasma GCs after injury. However, heart rate in particular is also elevated by the benzocaine anesthetic, which may have unknown effects on blood glucose^56^.

### In vitro incubation of adrenal tissue identified distinct mechanisms for cortisol and corticosterone synthesis and release

**Fig. 6:**
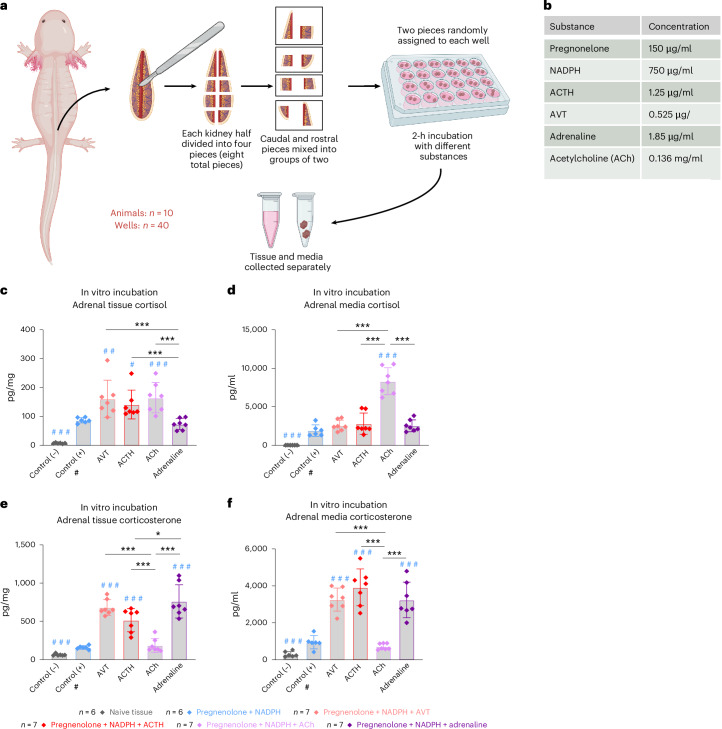
In vitro incubation of axolotl adrenal tissue with GC-stimulatory substances. **a**, Experimental design. Kidneys with adrenal tissue were collected from axolotls and trimmed to remove excess kidney tissue before dividing into eight pieces and randomly allocating groups of two non-horizontally adjacent pieces into a well plate with culture media. The adrenal tissue was then incubated with media only (negative control), pregnenolone and NADPH only (required for GC synthesis; positive control) or pregnenolone, NADPH and different stimulatory substances as listed in **b**. After 2 h of incubation, tissue and media were collected separately to obtain proxy measurements of both GC synthesis and release. All ten animals used for tissue collection were of mixed sex and 1–1.5 years old. **b**, Stimulatory substances and associated concentrations added to incubation media. **c**, Analysis of cortisol concentration in adrenal tissue after 2-h incubation indicated that AVT, ACTH and ACh were equally stimulatory on cortisol synthesis and all more potent than adrenaline. **d**, Cortisol concentration in culture media after 2h incubation period indicated that ACh was the most potent stimulus of cortisol release. **e**, Concentration of corticosterone in adrenal tissue after 2 h of incubation indicated that AVT, ACTH and adrenaline were all potent stimuli of corticosterone synthesis, while ACh was not. **f**, Culture media after 2-h incubation period indicated that AVT, ACTH and adrenalin were all potent stimuli of corticosterone release, while ACh was not. All statistical analysis performed as GMML-γ followed by Tukey post-hoc tests. *P < 0.05, **P < 0.005, ***P < 0.0001. All error bars indicate standard deviation of the data. A blue hash mark indicates a significant difference from the positive control, while and an asterisk indicates statistical significance compared with other groups. Note that, although not indicated on graphs, all treatments were significantly different from the negative control (*P* < 0.0001) in both tissue and media. Numbers of replicate wells for each condition were: negative and positive control *n* = 6, all other conditions *n* = 7. Complete results of statistical analyses are given in Supplementary Table 2. Panel **a** created with BioRender.com.

To further understand how the mechanisms governing cortisol and corticosterone synthesis and release may be regulated independently, we performed an in vitro assay where adrenal tissue was incubated for 2 h with different potential stimulatory factors (Fig. 6a,b). Tissue and media were then assayed separately for cortisol and corticosterone content. All conditions were compared with each other and with the positive control wells in which only pregnenolone and NADPH were added as these are the necessary precursors for GC synthesis. All statistical comparisons for this experiment were performed by GLMM-γ and Tukey post-hoc tests.

For cortisol in tissue, the addition of AVT, ACTH and ACh increased tissue concentration compared with the positive control (GLMM-γ and Tukey post-hoc test; *z*(−4.106), *P* = 0.0006); *z*(−3.253), *P* = 0.0145 and *z*(−4.332), *P* = 0.0002, respectively), while adrenaline did not (Fig. 6c). When comparing the different treatments against each other, no difference was observed between the three factors, indicating that AVT, ACTH and ACh were equally potent in stimulating cortisol synthesis in the in vitro tissue samples. However, when assaying the media as a proxy of cortisol release (Fig. 6d), only the ACh condition released more cortisol into the media than the positive control (GLMM-γ and Tukey post-hoc test *z*(−9.047), *P* < 0.0001). When comparing all treatments against each other, media cortisol was also significantly higher with ACh treatment compared with ACTH, AVT and adrenaline (GLMM-γ and Tukey post-hoc test; *z*(−6.972), *P* < 0.0001); *z*(7.488), *P* < 0.0001; *z*(−7.540), *P* < 0.0001), respectively), indicating that ACh was the most potent stimuli for cortisol release into the media.

When assaying corticosterone in the tissue samples, AVT, ACTH and adrenaline all significantly increased corticosterone concentration compared with the positive control (GLMM-γ and Tukey post-hoc tests *z*(−10.126), *P* < 0.0001 for AVT; *z*(−8.074), *P* < 0.0001 for ACTH and *z*(10.838), *P* < 0.0001 for adrenaline), while ACh did not (Fig. 6e). When comparing all the treatments against each other, the tissue concentrations of corticosterone were all significantly higher with AVT, ACTH and adrenaline compared with ACh (GLMM-γ and Tukey post-hoc tests *z*(−9.636), *P* < 0.0001 for AVT; *z*(7.526), *P* < 0.0001 for ACTH and *z*(10.385), *P* < 0.0001 for adrenaline). Furthermore, adrenaline led to higher tissue concentrations than ACTH (*z*(−2.869), *P* = 0.0473), but not AVT. For corticosterone in the media, all conditions also led to significantly higher media concentration compared with the positive control (*P* < 0.0001), except for ACh, which again did not influence media corticosterone in this setting (Fig. 6f). When comparing the treatments, there was no difference between AVT, ACTH and adrenaline, but all three produced media concentrations significantly higher than ACh (GLMM-γ and Tukey post-hoc tests *z*(−9.622), *P* < 0.0001 for AVT; *z*(10.800), *P* < 0.0001 for ACTH and *z*(9.548), *P* < 0.0001 for adrenaline). Finally, when comparing the tissue concentrations of cortisol and corticosterone, corticosterone was higher than cortisol in all conditions including the positive control (GLMM-γ and Tukey post-hoc tests *z*(4.917), *P* < 0.0001 for the positive control; *z*(12.890), *P* < 0.0001 for AVT; *z*(11.407), *P* < 0.0001 for ACTH and *z*(20.526), *P* < 0.0001 for adrenaline), except for ACh where the difference was no longer significant (Supplementary Fig. 1g). In the media, there was also a significant difference between cortisol and corticosterone in the positive control, with cortisol being higher when no stimulatory factor was added (GLMM-γ and Tukey post-hoc test *z*(−4.672), *P* < 0.0001) (Supplementary Fig. 1h). However, due to the more potent effect on corticosterone release than cortisol release by AVT, ACTH and adrenaline, there was no longer a significant difference between cortisol and corticosterone in the AVT and adrenaline groups, while corticosterone was higher when ACTH was added (*z*(2.533), *P* = 0.0113). In stark contrast to these effects, ACh led to a much higher release of cortisol than corticosterone (*z*(−17.682), *P* < 0.0001) (Supplementary Fig. 1h).

Overall, these in vitro experiments demonstrated that axolotl adrenal tissue synthetized higher levels of cortisol in response to AVT, ACTH and ACh but not adrenaline and that, out of all the factors tested, ACh was the most potent in stimulating cortisol release into the media (Fig. 6c,d and Supplementary Fig. 1g,h), supporting the hypothesis that the release of cortisol could be stimulated independently of the HPI axis, via direct sympathoadrenal nerve signaling. A similar response to ACh, only with corticosterone, has been documented in isolated frog adrenals^57^.

For corticosterone, ACh did not stimulate its synthesis or release, whereas AVT and ACTH were both potent stimuli, suggesting that corticosterone responses are primarily regulated by the classical HPI-axis pathway rather than direct neural signaling (Fig. 6e,f and Supplementary Fig. 1g,h). Adrenaline also had a significant effect on corticosterone, which indicates that there may be important crosstalk between the catecholamine and GC system. However, as previously shown, the systemic elevation in adrenaline level after stress in vivo was very rapid, but brief (Fig. 4j). This interaction with adrenaline might explain why corticosterone increased earlier than cortisol after manual stress and amputation (Figs. 4 and 5).

### The axolotl adrenal contains a heterogeneous population of GC-producing cells

**Fig. 7:**
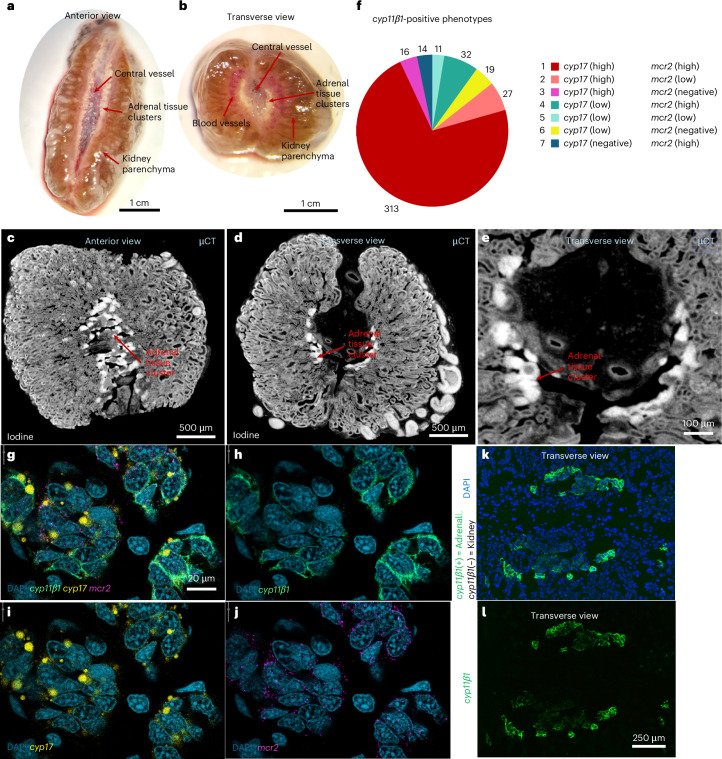
Tissue morphology and CYP11β1-positive cell phenotypes in the axolotl adrenal. **a**, Image of the freshly excised kidney from a male animal (56 g body mass, approximately 1.5 years old) in anterior view. The kidney parenchyma appears as brown tissue, while adrenal tissue is identifiable as medial yellow–golden clusters distributed along the central vessel. **b**, The same specimen seen in **a**, cut across the transversal plane to show the distribution of adrenal clusters in a circular pattern around the central vessel. **c**, µCT image of the same specimen shown in **a** and **b** after contrast staining in iodine, here shown in anterior view (as in **a**) with adrenal clusters recognizable as clusters of cells with higher affinity for iodine compared with the kidney parenchyma. **d**, µCT image of the same specimen shown in **a**–**c**, here in the transverse view (as in **b**). **e**, Zoomed view of the central vessel with surrounding adrenal clusters from **d**. The complete dataset from µCT imaging of the specimen is available via figshare at 10.6084/m9.figshare.30903434.v1 (ref. ^93^). **f**, Classification of subpopulations within the axolotl adrenal. Individual cells were identified on HCR in situ labeled cryosections (four sections each from five tissue samples/animals) and classified on the basis of the following criteria: negative = <10 puncta per cell; positive = minimum 10 puncta; low = between 10% and 25% of average number of puncta in channel; high = >25% of average number of puncta. **g**, HCR in situ composite image of all four channels. Single-plane confocal images obtained at 40× magnification. **h**–**j**, Same image as **g**, but with only DAPI (blue) and cholesterol side-chain cleavage enzyme P450scc mRNA (*cyp11β1*) (green) (**h**), cytochrome P450 family 17 mRNA (*cyp17*) (yellow) (**i**) or *mcr2* mRNA (magenta) (**j**) displayed. **k**, Widefield fluorescent image obtained at 20× magnification showing adrenal tissue (*cyp11β1* positive/green) within kidney section with DAPI (blue) nuclear stain. **l**, Same image as **k** with *cyp11β1* (green) without DAPI. All animals used for HCR were of mixed sexes and 1 year old.

Due to the distinct differences in the dynamics of cortisol and corticosterone synthesis and release in response to different kinds of stress activation and stimuli, we wanted to examine whether this could potentially be due to distinct steroidogenic cell populations producing cortisol and corticosterone respectively. Anatomical depictions of the tissue are shown with photographs and micro-computed tomography (µCT) images in Fig. 7a–e. We performed fluorescent hybridization chain reaction (HCR) in situ staining against transcripts for cholesterol side-chain cleavage enzyme P450scc (CYP11β1), cytochrome P450 family 17 (CYP17) and MCR2 mRNA transcripts on adrenal tissue sections (Fig. 7f–l). CYP11β1 is an enzyme required for all GC synthesis and is thus a positive marker for steroidogenic cells in general, while CYP17 is required for the final step in cortisol synthesis (for instance, most rodents produce mostly corticosterone due to low expression of CYP17^58^). MCR2 is the ACTH receptor.

While most of the *cyp11β1*-positive cells (steroidogenic cells) were also highly positive for both *cyp17* and *mcr2* (population 1; Fig. 7f), smaller populations were also found with differing expression levels, including a small population of cells highly positive for *cyp17* with low expression of *mcr2* (population 2) as well as another highly positive for *cyp17* and negative for *mcr2* (population 3). These subpopulations could represent cells producing cortisol independently of ACTH/MCR2 signaling. Also, smaller populations of *cyp11β1*-expressing cells were found to be *cyp17* (low)/*mcr2* (high) (population 4) and *cyp17* (negative)/*mcr2* (high) (population 7), which would be cells capable of producing corticosterone, but not equivalent amounts of cortisol, under ACTH regulation. Widefield fluorescent images show the overall distribution of *cyp11β1*-positive cells within the kidney/adrenal tissue (Fig. 7k,l).

These results show that the axolotl adrenal tissue contained a heterogeneous population of steroidogenic interrenal cells, which in part could explain how cortisol and corticosterone are regulated via different mechanisms.

### Upon i.v. injection of cortisol and corticosterone, only cortisol reduced glucose uptake in skeletal muscle and liver

**Fig. 8:**
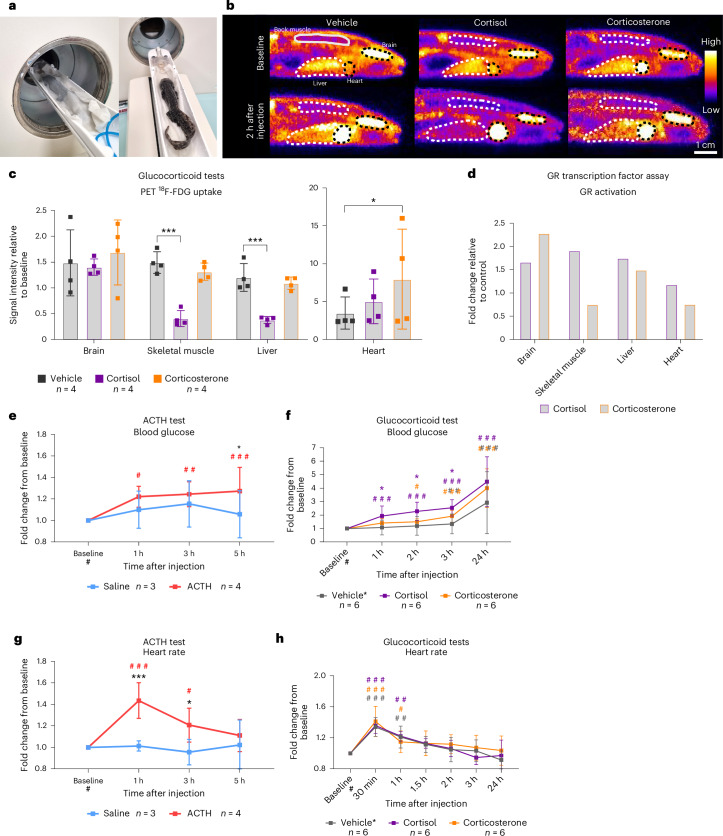
In vivo response to GC signaling. **a**, Placement of axolotl in the scanning bed of the PET scanner (left) and axolotl fully covered in moist paper towels inside the scanner positioned for imaging (right). **b**, Representative images of sagittal slices of PET images of ^18^F-FDG (glucose analog) distribution 2 h after tracer injection and before GC injection (baseline) and 2 h after administration of vehicle (DMSO), cortisol or corticosterone to quantify glucose uptake resulting from GC stimuli. **c**, PET signal in regions of interest relative to steady-state (pre-GC injection). Vehicle *n* = 4, cortisol *n* = 4 and corticosterone *n* = 4. **d**, GR activation measured by GR response element transcription activation assay on pooled samples of nuclear extracts from different tissues. Values on the *y* axis are the fold change from measurements on untreated samples. Brain tissue samples were incubated with 1.5 ng/ml cortisol or corticosterone, while other tissues were treated with 7.5 ng/ml. **e**, Blood glucose measured with a glucometer as fold change compared with baseline after injection with ACTH or saline. Saline *n* = 3, ACTH *n* = 4. **f**, Blood glucose measured with glucometer as fold change relative to baseline in animals injected intravenously with vehicle (DMSO), cortisol or corticosterone. Vehicle *n* = 6, cortisol *n* = 6 and corticosterone *n* = 6. **g**, Heart rate measured by echocardiography as fold change from baseline after i.m. injection with ACTH or saline. Saline *n* = 3, ACTH *n* = 4. **h**, Heart rate measured by echocardiography as fold change relative to baseline in animals injected intravenously with vehicle (DMSO), cortisol or corticosterone. Vehicle *n* = 6, cortisol *n* = 6 and corticosterone *n* = 6. Statistical significance was determined by generalized linear mixed modeling with gamma distribution and Šidák (comparison with baseline (#)) or Tukey post-hoc tests (multiple or single pairwise comparison between groups (*)). *P < 0.05, **P < 0.005, ***P < 0.0001. All error bars indicate the standard deviation of the data. Squares in bar graphs represent individual replicates. Asterisks indicate statistical significance between saline and ACTH groups and hash marks indicate significance between baseline and other time points within a group. All groups were of mixed sex, and animals were 1.5–2.0 years old. Complete results of statistical analyses are given in Supplementary Table 2.

To further explore the dynamics of the in vivo response to cortisol compared with corticosterone, we injected axolotls with fluorodeoxyglucose (^18^F-FDG), a radioactive glucose analog used as a positron emission tomography (PET) radiotracer as a proxy for glucose uptake. Two hours after i.v. injection of ^18^F-FDG, we imaged the axolotls for the first time in the PET scanner (Fig. 8a) to obtain a baseline image at the steady state level of ^18^F-FDG uptake and decay (Fig. 8b, top) before injecting them with vehicle (dimethyl sulfoxide (DMSO), a GC solvent), cortisol or corticosterone. After another 2 h of circulation time with vehicle/GCs, the animals were PET-scanned again (Fig. 8b, bottom) and ^18^F-FDG activity was measured in target tissues. In a perfect steady-state situation, the ratio of ^18^F-FDG activity at 2 and 4 hpi would be 1, when adjusting for the exponential decay of ^18^F with a known half-life of 109.7 min. A ratio above 1 means steady state was not completely achieved at 2 hpi, and a ratio below 1 means a decrease in ^18^F-FDG uptake in the 2 hpi period between the first and second PET scan when vehicle/GCs were circulating. All statistical comparisons for this experiment were performed by GLMM-γ and Šidák (comparison within a group across time) or Tukey (comparison between groups) post-hoc tests.

In neural tissue (brain, eye and olfactory lobes), we observed that steady state was not quite achieved after 2 hpi (signal ratio ~1.5), but there was no effect of any GC treatment relative to vehicle (Fig. 8c and Supplementary Fig. 3). Cardiac uptake was not in steady state (signal ratio of 4–8), but there was a significant increase in glucose uptake compared with vehicle in the corticosterone-treated animals only (GLMM-γ and Tukey post-hoc test; *z*(2.998), *P* = 0.0054). In skeletal muscle, gills and liver, ^18^F-FDG uptake/decay at 2 hpi was very close to steady state (signal ratio of 1) (Fig. 8c and Supplementary Fig. 4). Interestingly, following cortisol injection, the uptake of ^18^F-FDG decreased significantly in skeletal muscle and liver compared with vehicle (GLMM-γ and Tukey post-hoc tests; *z*(−5.172), *P* < 0.0001 for skeletal muscle and *z*(−4.505), *P* < 0.0001 in liver) (Fig. 8c). Reduced uptake of glucose in these tissues is a well-known effect of GCs in other species used as a mechanism to maintain high blood glucose by decreasing uptake into skeletal musculature and liver while increasing endogenous glucose production in the liver from glycogen breakdown^59^. In a GR transcription factor activation assay, which measures the level of GC bound receptors (indication of ongoing GC signaling), we also observed different effects of cortisol and corticosterone on a range of tissue samples in vitro (Fig. 8d), although statistical analysis could not be performed as each replicate was pooled from six samples.

### Blood glucose was elevated by ACTH, cortisol and corticosterone, with cortisol being more potent than corticosterone

Elevated blood glucose is, in conjunction with the decreased uptake of glucose in muscle and liver, a classical downstream effect of GC signaling^59^. During an ACTH test, while there was no statistically significant increase from baseline in blood glucose of saline-injected animals, blood glucose levels were different from baseline in ACTH-injected animals at 1 hpi, 3 hpi and 5 hpi (GLMM-γ and Šidák post-hoc tests *z*(3.696), *P* = 0.0009 at 1 hpi; *z*(3.968), *P* = 0.0003 at 3 hpi and *z*(4.362), *P* = 0.0001 at 5 hpi) (Fig. 8e). When comparing the two groups, a statistically significant difference could be observed at 5 hpi (GLMM-γ and Tukey post-hoc test *z*(2.337), *P* = 0.0194). Next, we measured blood glucose levels after the GC test (Fig. 8f) and found that, while there was an increase compared with baseline in all groups (probably a handling effect during imaging), only cortisol produced an effect that was significantly higher than the vehicle group at 1 hpi, 2 hpi and 3 hpi of GCs (GLMM-γ and Tukey post-hoc tests *z*(2.608), *P* = 0.0181 at 1 hpi; *z*(2.456), *P* = 0.0088 at 2 hpi and *z*(2.390), *P* = 0.0334 at 3 hpi). This observation supports our finding with PET imaging that cortisol had a greater effect on physiological glucose.

### ACTH but not GCs increased heart rate

Elevated heart rate (and cardiac metabolism) is another well-known effect of GC signaling^60,61^. Heart rate increased significantly after ACTH injection from baseline at 1 hpi and 3 hpi (GLMM-γ and Šidák post-hoc tests; *z*(5.718), *P* < 0.0001 at 1 hpi and *z*(2.929), *P* = 0.0135 at 3 hpi) (Fig. 8g). The difference between the saline and ACTH group was also significant at 1 hpi and 3 hpi (GLMM-γ and Tukey post-hoc tests *z*(3.870), *P* < 0.0001 at 1 hpi and *z*(2.627), *P* = 0.0086 at 3 hpi). After the GC test, heart rate increased from baseline in all groups including vehicle, but neither cortisol nor corticosterone produced an effect significantly different from the vehicle control (Fig. 8h). Altogether, the results from the GC tests indicate that, in terms of the typical effects on blood glucose and reduced glucose uptake in skeletal muscle and the liver, cortisol was more potent, although both GCs showed a similar tendency; corticosterone was more effective in increasing cardiac glucose uptake. While ACTH increased heart rate, the same effect was not seen with direct stimuli with GCs.

## Discussion

While we set out with a primary simplistic goal of defining the dominant GC in the axolotl, as has been reported for many other species previously, we found an unexpectedly complex GC response involving both cortisol and corticosterone, activated differently depending on the type of stressor involved. When evaluating the results of this study, we conclude that axolotls can activate the classical GC pathway via AVT and ACTH, especially upon pharmacological intervention with both AVT (Fig. 3) and ACTH (Fig. 2) and directly after amputation injury (Fig. 5), most prominently prompting corticosterone synthesis and release. There also appears to be a crosstalk between the HPI axis/corticosterone and catecholamines given that adrenaline could, similarly to ACTH, prompt corticosterone synthesis and release (Fig. 6e,f). We have not tested whether combining ACTH and adrenaline would have an additive effect, but the faster increase in corticosterone compared with cortisol after manual stress (Fig. 4) and amputation (Fig. 5b,c) could potentially be a result of adrenaline, which we saw was released into the circulation rapidly after manual stress (Fig. 4j). An important implication of these findings is that adrenergic signaling, which has been reported to involve a systemic activation response following limb regeneration, priming axolotls for further regeneration and, thus, leading to faster regeneration of other body parts^62^, will probably lead to a downstream release of GCs. This release of GCs has a substantial impact on animal physiology and has largely unknown direct effects on tissue regeneration. It is not known if GC similarly instigates a systemic activation response, and the effects of GC on regenerative processes in axolotl warrant future investigation. The direct stimulatory effect on cortisol release by ACh (Fig. 6) also opens the possibility of novel levels of interplay between nerve signaling, catecholamines, GCs and regenerative progression. Interestingly, in the context of axolotl spinal cord regeneration, a specific group of neurons in the axolotl brain has been shown to be activated upon injury and involved in driving the regenerative progress via a mechanism involving the hypothalamus and neurotensin and ultimately promoting the release of growth hormone^63^. Growth hormones are, in turn, well known to be regulated by GCs^64^ as well as ACTH^65^.

In contrast to the dynamics of corticosterone, cortisol was more prominent after the manual stress test (Fig. 4) but was also significantly upregulated after amputation performed under anesthesia (Fig. 5) (although to a lesser extent than corticosterone). It is unclear at this time to what extent the unanesthetized perception of stress or pain versus physical insult/injury independent of perception would contribute toward the GC response in an animal model like the axolotl. Due to ethical guidelines and considerations, it is not possible or desirable to induce this kind of injury in an unanesthetized animal. We also found that cortisol activation occurred largely via a nonclassical mechanism, as inhibiting ACTH signaling via the MCR2 receptor did not significantly impact the synthesis or release of cortisol, while corticosterone was indeed diminished by the same intervention (Fig. 4). Furthermore, we observed that the most potent stimuli of cortisol release tested in vitro was ACh, and not ACTH or AVT, although these also had a stimulatory effect on release and an equivalent effect on synthesis (Fig. 6). These results point to an ability of sympathoadrenal nerve signaling to directly stimulate cortisol release in the axolotl interrenal tissue.

Importantly, the existence of pathways capable of inducing GC release independent of ACTH is not an entirely novel concept. Several effectors have been shown to engage in ACTH-dissociated GC release in other species, including several neuropeptides, neurotransmitters, opioids, growth factors and inflammatory cytokines; adrenocortical cells were also found to express a plethora of receptors capable of binding these non-HPI axis factors, as more extensively reviewed by Bornstein et al.^66^. In addition, there is increasing evidence for a local intra-adrenal/-renal regulation of GC synthesis and release independent of ACTH^67–69^. For instance, a previous study on in vitro preparations of axolotl interrenal tissue also showed that ACTH and AVT could promote the release of corticosterone involving regulatory effects distinct from anurans and that this release was regulated in part by Ca^2+^ ions^70^. AVT has also been shown to induce the release of corticosterone from xenopus interrenal tissue^71^. In both cases, cortisol was not assayed. It is, however, still surprising to find that ACh is a more potent initiator of cortisol release than ACTH in the axolotl (Fig. 6). This result suggests that physical stress induces the release of cortisol more so via sympathoadrenal nerve signaling, which is classically attributed to the regulation of catecholamines rather than GCs; nervous supply to the chromaffin tissue in amphibians has also classically been thought to be limited^72^.

This is especially relevant because cortisol is the GC that better fits the profile of an endogenous GC in the axolotl compared with corticosterone. This is evident by its prominent activation via manual stress rather than pharmacological manipulation and its downstream effector responses seen on blood glucose concentration and tissue uptake of glucose; however, the heart seems to be more sensitive to corticosterone (Fig. 8). A future avenue of research could be to directly examine the colocation and the potential for crosstalk between populations of steroidogenic and chromaffin cells in the axolotl adrenal as has been reported in the newt (*Triturus carnifex*)^73^, as well as the implications of the presence of different populations of steroidogenic cells that we identified on the basis of their expression of *cyp11β1*, *cyp17* and *mcr2* mRNA (Fig. 7). The different timescales and levels of plasma concentrations of catecholamines recorded here in axolotls after manual stress, with plasma noradrenaline seemingly increasing later and to a lesser extent than adrenaline and dopamine (Fig. 4), could also be interesting to study in this context, as in the newt, dopamine and epinephrine were inhibitory to steroidogenic tissue, whereas noradrenaline was stimulatory^73^.

In this study, we opted to administer AVT as the upstream instigator of ACTH based on previous reports that AVT is the primary stimulator of the HPI axis in amphibians^74^. It is unclear what role CRH plays in activating stress responses, or how the resulting crosstalk between AVT, CRH and the HPI versus HPT axis might affect axolotls. This has important implications for axolotls due to their pedomorphic nature; whether axolotls use inhibitory regulation of CRH and/or ACTH, with concurrent effects on glucocorticoids, to prevent spontaneous metamorphosis represents an important avenue for further investigation. Despite an extensive body of research, the exact mechanisms that maintain pedomorphy are not fully understood^27,28,75^. GCs have been demonstrated to be involved in metamorphosis; in amphibians that undergo normal metamorphosis, the process is regulated by the HPT and HPI axes^23–26^. Previous studies have demonstrated how these two systems act in concert to facilitate successful metamorphosis, as reviewed most recently by Sachs et al.^26^. Genetic knockout models and thyroidectomized frogs have demonstrated that, while the HPT axis alone can induce most of the morphological changes associated with metamorphosis, GC signaling is required to complete metamorphosis and ensure long-term survival post-metamorphosis^76–78^. The tight relationship between the HPI and HPT axes and the pro-metamorphic effects of GCs may demand a tight regulation of the upstream elements such as AVT and ACTH in pedomorphs and may have driven the development of divergent roles for cortisol and corticosterone. Maintaining neoteny may require a distinction between glucocorticoid signaling associated with exposure to mild, common stressors (primarily via cortisol as an extension of the adrenal stress response) and that associated with prolonged and/or severe stressors that activate the HPI axis (via corticosterone), which risks instigating metamorphosis. Here, we are limited in our understanding of potential links to pedomorphy; to elucidate this issue, it would be necessary to repeat some of these experiments in metamorphosed axolotls, although this study would be complicated by the involution of the external gills eliminating access to the vessels that we have used to collect blood samples from neotenic animals (Supplementary Fig. 2). In addition, because metamorphosis in axolotls is typically induced pharmacologically by administering synthetic thyroid hormone, it is difficult to know with certainty whether the resulting phenotype is truly equivalent to spontaneous metamorphosis induced by environmental stress (which almost certainly involves stress hormones in addition to thyroid hormones). Inducing spontaneous metamorphosis by, for instance, lowering the housing water for an extended period, mirroring now infamous experiments from the 1800s^79^, would require critical ethical considerations. The closest approximation could involve using closely related species, such as tiger salamanders, that naturally undergo metamorphosis.

**Fig. 9:**
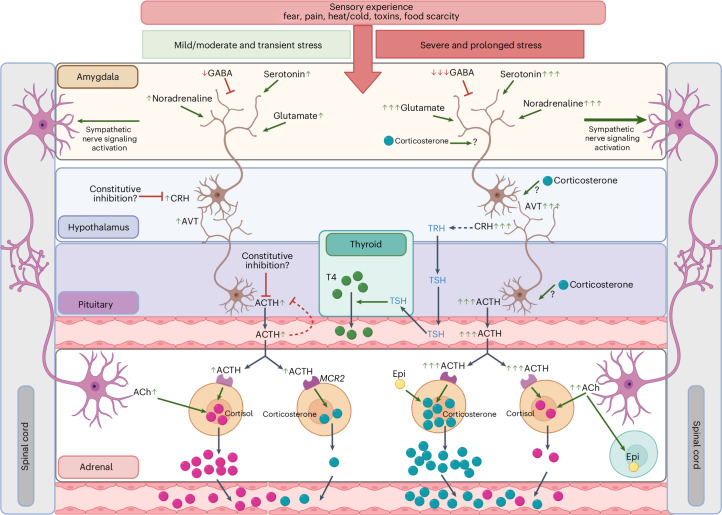
Potential mechanisms underlying differential response to mild versus severe stress. The figure shows proposed mechanisms that could be involved in distinct dynamics of cortisol and corticosterone under different degrees of stress. Under this hypothesis, mild/moderate and transient stress leads to a cortisol-dominant response, primarily driven by sympathetic nervous system activation (possibly due to constitutive inhibition of upstream signals such as CRH and AVT from the hypothalamus, or ACTH from the pituitary). Sympathetic nerve signaling triggers acetylcholine release, inducing not only the secretion of catecholamines such as adrenaline (Epi) from the adrenal gland, but also cortisol. Conversely, severe and/or prolonged stress activates the HPI axis, which ultimately leads to the binding of ACTH to MCR2 and the release of corticosterone, which could crosstalk with the HPT axis at multiple points (such as effects on upstream neurotransmitters, thyrotropin-releasing hormone (TRH) and thyroid-stimulating hormone (TSH)), which could contribute to the onset of metamorphosis, a mechanism that is normally strongly inhibited in order to maintain neoteny in the axolotl. Small green and red arrows next to the factor names indicate whether these factors are present at higher or lower levels compared with homeostasis. Elongated green arrows between factors and neurons indicate a stimulatory effect, red lines indicate inhibitory effects and black arrows indicate the movement of a factor from one compartment to another. Figure created with BioRender.com.

We put forward a novel hypothesis (Fig. 9) in which axolotls rely on cortisol as their dominant GC when exposed to mild, moderate or brief stressful conditions, acting in continuation of the catecholamine system, thereby reducing the need for AVT and/or ACTH signaling that might risk activating the HPT axis, which would not be beneficial to maintaining pedomorphy. When an axolotl encounters severe or persistent stress, such as injury (here modeled by limb amputation under anesthesia), we propose a mechanism in which an inherent inhibition of the HPI axis is lifted via a currently uncharacterized process, thereby inducing AVT and/or CRH, ACTH and corticosterone, which may be required to elicit a robust and sufficient behavioral and endocrinological response. Likewise, HPI-axis activation could have an important and currently underappreciated role in mounting the necessary response associated with regeneration after injury. Finally, when axolotls are exposed to extreme and prolonged severe stress, HPI-axis activation and increased levels of corticosterone could result in HPT-axis activation, instigating spontaneous metamorphosis. To generalize this hypothesis across injury modalities, similar experiments would need to be performed across multiple injury sites and severities. To this point, systemic activation driven by adrenergic signaling following limb activation has been shown to happen only when the injury is substantial enough^80^. Also relevant here is the fact that repeated or chronic injury stimuli eventually lead to regenerative failure, with tolerance to elevated stress hormones being a possible mechanism^81^. In zebrafish, GC signaling impairs fin regeneration^82–84^, and the regeneration-inhibitory effects of GC treatment in larvae can persist through adulthood^85^. Thus, it will be important and exciting to dissect how the balance between adrenergic signaling, with its short-term regenerative benefits, might interface with GC signaling in the axolotl.

Several factors contributing to the distinct features of cortisol and corticosterone in axolotls remain to be explored, including a more detailed phenotyping of the different populations of cells within the adrenal tissues (Fig. 7), potential distinct binding specificities or expression profiles of receptors^86^, chaperones and plasma proteins, and the presence of different receptor variants, including both cytosolic and membrane-bound isotypes. Regulation by steroid-binding proteins is not well understood but could represent a key mechanism for both limiting the availability of free GCs and delivering steroids to specific target tissues^87^. This could, for instance, explain why corticosterone is seemingly more capable of activating the GR receptor in the brain when cortisol is more potent in other tissues (Fig. 8d). A negative feedback loop on certain elements of the HPI axis could also be part of the explanation of how manual stress could result in a completely different GC response in comparison with direct pharmacological intervention.

By exploring available transcriptome data^88^, we were able to identify several GC-related genes in the axolotl with varying expression levels in different tissues (Supplementary Fig. 4). A more detailed description of these genes is found in Supplementary Table 1. Different expression levels of these chaperone proteins that interact with the GR and GCs could also underly the regulatory mechanisms that underpin different responses to cortisol and corticosterone locally. The possible downstream regulatory effects represent an important avenue for further research. This is exemplified by a 2019 study reporting wild populations of salamanders having divergent transcriptomic responses to GCs depending on temperature^89^.

One important confounding factor not mentioned thus far in this study is the difficulty of fully accounting for the effects of circadian rhythm. We designed our experiments by mixing the groups so that, for example, saline blood samples were not all collected before ACTH blood samples, thereby minimizing this effect. The variance in baseline levels between different experimental rounds could very well reflect seasonal GC regulation (our experiments were performed at different times of the year during a 3-year period). Furthermore, there is probably some batch variation in baseline levels of GCs between the different experiments. Others looking to include assessments of GC and/or catecholamines in their studies should be aware of the importance of circadian rhythm and seasonal regulation for all adrenal hormones and catecholamines in amphibians^73,90–92^. How this effect plays out in controlled laboratory environments warrants further investigation.

Whether the divergent roles and mechanisms of cortisol and corticosterone are unique to the axolotl requires further study. When applying the axolotl as a model organism in any research field where stress hormones would be of interest as key signaling factors, it is clearly important to consider that cortisol and corticosterone may be acting independently and in concert with different downstream effects.

It is also possible that the divergent regulation of cortisol and corticosterone is a feature of aquatic salamanders or, perhaps most intriguingly, a feature of pedomorphic salamanders. While the standard method for induction of metamorphosis in the axolotl is via administration of thyroxin^28,29^, the involvement and potential benefit of GC signaling during metamorphosis should be further investigated. The fact that simultaneous treatment with dexamethasone reduced the required concentration of thyroxine to achieve metamorphosis in axolotls^18,19^, coupled with experiments in anurans showing that blocking of GC signaling during metamorphosis results in incomplete metamorphosis and drastically reduced viability of the adult animal^26,76–78^, raises the question of whether the standard method for metamorphosing axolotls is insufficient.

Finally, with the results of this study in mind, we strongly encourage the inclusion of both cortisol and corticosterone when assaying the stress response in axolotls (and possibly other amphibians), as different injury models or interventions may produce different levels of both with distinct downstream effects.

## Methods

### Animals and housing

In total, 120 adult axolotls (>1 year old, ∼50 g body mass) were used in this study. Sex was determined by body shape and appearance of the cloaca, and animals assigned to mixed-sex groups aiming for a 50/50 mix of males and females as far as possible. All animals were housed individually in plastic crates, each with a hide large enough to cover the animal, and maintained on a 12-h light/12-h dark cycle at 20 °C in tap water. Animals were fed axolotl pellets (Axobalance, Aquaterratec) three times a week. At least 24 h before any induced stress, animals were fasted and moved from the animal facility to the experimental lab, to acclimatize before stress induction. All experiments and housing conditions were approved by the Danish National Animal Experiments Inspectorate (protocol #2020-15-0201-00688). Animals were not randomized. Animals from the same batch were used for the same experiments but not across different experimentations. During stress tests, injury and any experiment including administration of any active compound, the order of animals treated/stressed was such that control and noncontrol animals were used in a staggered order so that not all controls were treated in succession followed by noncontrols to minimize the effects of circadian rhythm. Humane endpoints were defined as a loss of more than 20% body mass, refusing food for more than 1 week or animals being nonresponsive to tail pinch. No animals were at any point excluded from the study or data analysis. The procedures carried out in this study were in accordance with the national Danish legislation for the care and use of laboratory animals.

### Anesthesia

For all blood sampling, we anesthetized the axolotls by adding 3.5 ml propofol (Propofol B, Braun) in a 10 mg/ml concentration per 1 liter housing water, 30 min before each handling (or 15 min before if propofol was given within the previous hour). We have previously reported the usefulness of propofol in axolotls^56,94,95^. Based on our previous findings, we applied propofol for this study when possible as the more commonly used benzocaine or tricaine (MS-222) has substantial effects on heart function and therefore may affect the activation of stress pathways and, thus, increase variability of GC concentrations. Importantly, propofol does not provide good analgesia and is therefore not appropriate for invasive procedures. Therefore, when collecting tissue samples or performing amputations, animals had to be anesthetized by submersion in benzocaine (Sigma-Aldrich, cat. no. B0600000) for 30 min (200 mg/l first dissolved in 3 ml acetone).

### ACTH stress test

Animals were given an i.m. injection of ACTH (Synacthen, CD Pharmaceutical) at 200 IU/kg (2 mg/kg) or a corresponding volume of saline (sterile axolotl Ringer´s solution; Supplementary Table 3) divided across four injection sites along the large dorsal epaxial muscles. Synacthen is a synthetic analog of the first 24 amino acids of human ACTH (the fraction of the full peptide required for full activity). Human ACTH(1–24) is 91.7% identical to axolotl ACTH (Supplementary Fig. 5). Blood was collected using heparin-coated 0.5 ml/30 G syringes from a gill artery (Supplementary Fig. 2). A blood sample was collected at baseline just before ACTH/saline injection, and 1 h, 3 h and 24 h after injection (Fig. 2a). Blood was then centrifuged for 5 min at 2,000*g*, and plasma was transferred to a cryovial, snap frozen and stored at −20 °C for 1 week before analysis. Initial pilot experiments were done to determine the appropriate times for blood sampling; as a result, alternative time points were excluded from the main experiment, with 10 min and 30 min showing no change from baseline and 5 h and 8 h showing a continued trend from 3 h toward a return to baseline by 24 h. After blood sampling, animals were placed on a cloth to remove surface water for 30 s. A Salivette swab for GC measurements (Sarstedt, cat. no. 51.1534.500) was then gently run across the axolotl snout to tail, along the lateral surface, five times on each side. Before use, each swab was cut into four pieces to fit in smaller vials. The swab was weighed before and after mucus collection to obtain mucus weight.

### AVT challenge test

A stock solution of AVT (arginine vasotocin trifluoroacetate salt, Cayman Chemicals, cat. no. CC-24768) was prepared in sterile axolotl Ringer´s solution at a concentration of 1 mg/ml. The solution was stored at −20 °C and thawed on ice and warmed to room temperature before use. An i.v. injection of AVT (1 µg/g body mass) was administered i.v. in the jugular vein (Supplementary Fig. 2). A corresponding volume of saline was given to the saline control group. Blood sampling was done at baseline just before injection and 10 min, 1 h, 3 h and 24 h after injection. We opted to include an earlier time point after AVT injection compared with ACTH as AVT was injected i.v. in the jugular vein to ensure it would reach the brain and would therefore be expected to have a more rapid effect compared with the i.m. injection of ACTH.

### Manual stress test

The manual stress test was performed twice on separate batches of animals to collect sufficient blood for both glucocorticoid and catecholamine assays (150 µl for GC and 150 µl for CA). In both cases, an initial baseline blood sample was first collected and an injection of saline or MCR2 antagonist (Ab Biotech, GPS1573 at 4 µg/g body mass)^96^, administered i.m. in the back muscles 30 min before initiating the stress test. For tissue collection, animals not exposed to manual stress but still injected with saline were included. In total, 2 h passed between the baseline blood sample and initiating stress, to allow the animals to first regain consciousness. To initiate the test, animals were placed on a rolling table in shallow water (5 cm depth). For 1.5 h, the table was forcefully shaken for 2.5 min every 5 min. At the end of the stress induction, the animals were lifted out of the water by hand ten times, to mimic the stress of transport and handling. Samples were collected after 1 h, 3 h, 5 h and 24 h, snap-frozen and stored at −20 °C for GC assays (Fig. 4a), and at 10 min, 1 h, 3 h, 5 h and 24 h for catecholamine assays. Time 0 was designated as the endpoint of the stress induction. Syringes used for collecting catecholamine samples were coated in EDTA instead of heparin owing to incompatibility of the assay and heparin-containing samples. Furthermore, EDTA and metabisulfite were added to the plasma samples to final concentrations of 1 mM and 4 mM respectively as directed in the catecholamine kit manual. Samples were centrifuged at 2,000*g* for 5 min, and the plasma was transferred to a fresh tube, snap-frozen in liquid nitrogen and stored at −80 °C for no more than 2 weeks before the assay.

### Amputation injury stress test

Due to the invasive nature of amputation injury, the animals were anesthetized with benzocaine during this test as required by the Danish National Animal Experiments Inspectorate (protocol no. 2020-15-0201-00688). To induce an equivalent level of surgical anesthesia at every blood sample time point, the animals were submerged in benzocaine (Sigma-Aldrich, cat. no. B0600000. 200 mg/l first dissolved in 3 ml acetone) for 30 min, with two exceptions: (1) between injury and the 10-min sample, animals were kept in moist paper towels out of the water, and (2) only a 15-min submersion was used before the 1-h sample to account for the remaining effects of the previous dosage. After an initial baseline sample, the animals were either exposed to a sham surgery where they were placed on the surgical table and the right forelimb touched with the surgical tools without performing an injury or amputated where the surgical scissors were used to sever the right forelimb halfway along the length of the humerus. The injury caused some blood loss, but all the animals survived the experiment. After injury, blood samples were collected at 10 min, 1 h, 5 h, 24 h and 96 h. In addition to collecting plasma samples for GC enzyme-linked immunosorbent assays (ELISAs), we also measured the animals’ heart rates at each time point using echocardiography (Vevo 2100 Imaging System, Visualsonics) by recording the time of five cardiac cycles, and measured blood glucose using a commercial glucometer device and measuring strips (Freestyle Precision Neo).

### Tissue sample collection and processing

After confirming the surgical level for terminal anesthesia with benzocaine, the animals were given an injection of heparin (50 µl of 5,000 IU/ml solution). Animals were then euthanized by exsanguination before collecting kidney/adrenal tissue and the brain, which were immediately snap-frozen in liquid nitrogen and stored at −20 °C for a few days. For brain tissue, the entire brain was collected by severing the olfactory and optic nerves as well as 1 mm caudal to the transition to the spinal cord, thus ensuring the inclusion of the pituitary gland. Because adrenal tissue is embedded within the kidney proximal to the central vessels, adrenal tissue cannot be easily collected in isolation. Therefore, the entire kidney was collected, and only the outer edges were trimmed away, leaving the central region containing adrenal tissue for downstream applications (in vitro assay and ELISAs). To prepare tissue lysates for ELISA assays, frozen samples of kidney/adrenal and brain were thawed on ice and homogenized in 500 µl ice-cold 70% phosphate-buffered saline (PBS) on a bead homogenizer (Beadbug 6, Benchmark Scientific) using vials filled with ceramic beads (five cycles of 30 s at 3,000 RPM). The samples were then sonicated for 30 s before 10-min centrifugation at 3,000*g* and 4 °C. The lysate was transferred to a fresh vial, with a 50-µl portion aliquoted for ACTH ELISA and the remaining sample extracted in diethyl ether for glucocorticoid assays.

### Diethyl ether GC extraction

Mucus samples were first eluted by adding 500 µl ice-cold PBS to each vial containing swabs, and the vials were placed on a thermoshaker at 500 RPM and 4 °C for 30 min. The swab was then removed. The same general extraction protocol was applied to eluted mucus samples, tissue lysates and media samples from the in vitro assay. A total of 500 µl diethyl ether was added to each sample. The vial was shaken for 30 s and left for 5 min to allow layers to separate. The organic upper phase was then collected into a fresh tube and the process was repeated twice more, adding 1,500 µl of diethyl ether in total and combining the organic layers. Diethyl ether was then evaporated to dryness in a vacuum centrifuge (20 min at 35 °C), and the sample was stored at −20 °C for a few days. On the day of the ELISA assays, samples were reconstituted in assay buffer (included in ELISA kits) and vortexed three times. Mucus samples were reconstituted in 250 µl, tissue samples in 1 ml and media samples in 1 ml.

### Cortisol and corticosterone competitive ELISA

Assays were performed as directed in the manufacturer´s protocols (Enzo Life Sciences, cat. nos. ADI-900-071 and ADI-900-097). In brief, samples were loaded in antibody-coated wells, incubated with conjugate and secondary antibody for 2 h, washed, and incubated with pNpp substrate for 1 h; finally, stop solution was added and the plate and absorbance was read at 405 nm. Plasma samples were thawed on ice before adding 1:100 steroid displacement reagent (included in ELISA kits). The assay was performed at room temperature and covered with foil during light-sensitive steps. A total of 30–40 µl plasma was loaded in each well; 100 µl mucus and tissue extract was loaded in each well; 90 µl of the media samples was loaded in each well for cortisol, and 30 µl for corticosterone. Both assays include a standard that was loaded at different concentrations with every run to determine the linear range and detection limits of the assay and perform the data analysis. The appropriate sample volume of each type was determined during a pilot run to ensure measurements within the standard curve range. Data analysis was performed as specified by the manufacturer by generating a standard curve and normalizing against the control wells (blank, total activity and nonspecific binding).

### ACTH competitive ELISA

The assay was performed according to the manufacturer’s protocol (MyBioSource, cat. no. MBS7606822). A total of 50 µl plasma samples, 10 µl kidney/adrenal tissue lysate or 15 µl brain tissue lysate was added to each antibody-coated well and incubated for 90 min at 37 °C. The plate was then washed and biotin-labeled antibody was added for a 1-h incubation at 37 °C, before another wash and 20-min incubation with 3,3’,5,5’-tetramethylbenzidine (TMB) substrate. Finally, stop solution was added and absorbance was read at 450 nm. The assay includes a standard that was loaded at different concentrations with every run to determine the linear range and detection limits of the assay and perform the data analysis. The appropriate sample volume of each type was determined during a pilot run to ensure measurements within the standard curve range. Data analysis was performed as recommended by generating a standard curve and normalizing against the blank wells.

### Catecholamine extraction and ELISA

Samples were extracted and assayed for catecholamines using a commercial kit (LDN 3-CAT High Sensitive Catecholamine ELISA, cat. no. BA E-5600R) according to the manufacturer’s manual. In brief, catecholamines were extracted from 150-µl samples using a premade *cis*-diol affinity gel plate, acetylated and then converted enzymatically and finally detected by competitive ELISA. A total of 30 µl of extracted sample was used for each assay well. The ELISAs included a standard that was loaded at different concentrations to determine the linear range and detection limits of the assay. Data analysis was performed as directed by the manufacturer.

### In vitro adrenal tissue incubation assay

The kidneys were collected from ten animals after terminal benzocaine anesthesia and processed on a cold metal plate placed on ice using scalpel. Kidneys were divided into four equal parts by sagittal cuts, which were further separated into two parts each (Fig. 6a). The eight tissue pieces were then recombined to mix caudal and rostral sections, accounting for potential differences in steroidogenic cell content along this axis. Two pieces were randomly assigned to each well of a 24-well plate containing cold 70% L15 medium (Sigma-Aldrich, cat. no. L4386). The random assignment was done to avoid the effect of time across the total 2-h tissue collecting procedure. The plate was kept at 4 °C while all samples were collected. Tissue pieces were then transferred to new 24-well plates with room-temperature 70% L15 medium with 5% serum (One Shot FBS, Thermo Fisher, cat. no. A3160402). Media also contained antibiotics in the form of 1 ml/100 ml of an antibiotic–antimycotic solution (Sigma-Aldrich, cat. no. A5955) and 120 µl/100 ml gentamicin sulfate-amphotericin (Lonza, cat. no. GA-1000). Next, the different prepared additives were added to each well as detailed in Fig. 6b, and the plate was incubated for 2 h at room temperature on a shaker set to 400 RPM. Afterwards, tissue and media were collected separately in cryovials and snap-frozen in liquid nitrogen. Tissue samples were weighed before freezing.

### GR activation assay

Tissue samples of ∼100 mg were incubated in a 24-well plate with 1 ml of 70% MEM media (Sigma-Aldrich, cat. no. M4655) with different concentrations of either corticosterone or cortisol (0, 1.5 or 7.5 ng/ml) for 1 h in an incubator set to 25 °C and 1.2% CO_2_. Tissues were removed from the media and washed in ice-cold PBS, then homogenized in an extraction buffer containing 0.1% dithiothreitol using a bead homogenizer. The supernatant was then used for nuclear extraction using a commercial kit (Abcam, cat. no. ab113474). Samples from the same treatment group (*n* = 6) were pooled and used for a colorimetric glucocorticoid receptor transcription factor assay kit according to the manufacturer’s manual (Abcam, cat. no. ab207207) as well as a reducing-agent-compatible BCA assay (Invitrogen, cat. no. 23250) to determine the total protein concentration. This assay measures the number of GRs bound by GCs at the time of sampling, thereby reflecting the level of GC signaling under different conditions or in different tissues

### In vivo administration and response to cortisol and corticosterone

GC solutions were prepared by dissolving 10 mg/ml cortisol (hydrocortisone, BioNordika Denmark) or corticosterone (BioNordika Denmark) in sterile DMSO and then further diluting the solution 1:4 with sterile axolotl Ringer’s solution to reach a final concentration of either compound of 2 mg/ml. Before injection, animals were weighted and cortisol, corticosterone or vehicle (1:4 solution of DMSO in axolotl Ringer’s solution) was intravenously injected into the jugular vein at a dosage of 7.14 mg/kg body mass, resembling a typical high dosage of 500 mg hydrocortisone in an average 70-kg human being. Heart rate was measured using a handheld fetal Doppler device (SonoTrax Basic A equipped with a 2-MHz transducer) across five cardiac cycles. Blood glucose was measured using a commercial glucometer (FreeStyle Precision Neo) by placing a single drop of blood on a measuring strip. For PET imaging, the animals were first injected with ^18^F-FDG (~25 MBq per animal), a radioactive glucose analog with a half-life of 109.8 min, which was allowed to circulate for 2 h before we administered GCs. The 2-h ^18^F-FDG circulation time was based on previous PET-imaging experiments in axolotl on the time to reach a steady state of ^18^F-FDG uptake and decay^97^. After 2 h, the animals were PET-scanned to obtain a baseline image of steady state ^18^F-FDG (that is, glucose uptake) and then injected with either vehicle, cortisol or corticosterone (7.14 mg/kg body mass). Another 2 h after injecting GCs, the animals were imaged in the PET scanner again before being returned to their housing water. PET imaging was performed using a Mediso NanoScan PET/MRI system set to detect ^18^F decay with an isotropic image resolution of 0.4 mm and an acquisition time of 10 min per scan. During PET imaging, the animals were wrapped in moist paper towels. All acquired PET images were adjusted for the exact radioactivity of injected ^18^F-FDG and the radioactive decay between the time of ^18^F-FDG injection and the time of imaging. To quantify any changes from steady state uptake of ^18^F-FDG in different organs and anatomical structures, similar sized regions of interests were placed at the brain, eye, olfactory lobes, gills, heart, liver and back muscle. The ^18^F-FDG-activity ratio at these regions after vehicle/cortisol/corticosterone circulation relative to the baseline scan was calculated to quantify whether glucose uptake was affected by any of the GCs.

### HCR RNA fluorescence in situ hybridization

Kidneys with adrenal tissue were collected and fixed in MEMFA buffer with 3.7% paraformaldehyde overnight at 4 °C. After washing with PBS, samples were equilibrated in 15% sucrose for 6 h, followed by 30% sucrose overnight, before being embedded in OCT cryoembedding medium. The frozen blocks were then sectioned at 8 µm thickness and stored at −80 °C for a few days. To perform HCR, we used the protocol and probe design tool established by Lovely et al.^98^ using commercial reagents from Molecular Instruments. The custom-designed probes against axolotl cholesterol side-chain cleavage enzyme P450scc (*cyp11β1*) mRNA, cytochrome P450 family 17 (*cyp17*) mRNA and *mcr2* mRNA were purchased from Integrated DNA Technologies. In brief, sections were cleared using a boric-acid-based solution, followed by incubation with hybridization buffer and HCR probes overnight. The next day after a series of washes, slides were incubated with HCR amplification buffer and the HCR hairpin solution prepared on a thermocycler and added to the sections. Finally, sections were counterstained with DAPI nuclear stain and mounted with aqueous mounting medium. Images were acquired on a Zeiss LSM 900 Confocal system at 40× magnification and analyzed in Qupath by combining the ‘Cell detection’ and ‘Subcellular detection’ features. Quality controls were performed by omitting the probes to test for unspecific signal from the hairpins alone, which yielded no detectable signal with confocal imaging. Furthermore, none of the adrenal-specific probes yielded positive staining in the surrounding kidney tissue, as seen in Fig. 7.

### µCT imaging

An intact kidney with adrenal tissue was excised from a euthanized adult male axolotl (56 g body mass, approximately 1.5 years old). The central portion (most caudal and rostral portion removed) was imaged under the stereomicroscope and then immediately transferred to 4% paraformaldehyde and fixated for 3 days at 4 °C. Following fixation, the sample was washed in three changes of 70% PBS over the course of 1 h before being placed in 1.25% Lugol’s iodine solution (4.17 g/l I_2_ and 8.33 g/l KI) for 3 days at room temperature with gentle agitation. After staining with iodine, the sample was again washed in three changes of 70% PBS before being stored in 4% formaldehyde at 4 °C for a few days. For imaging, the specimen was mounted in a P1000 pipette tip using small pieces of Styrofoam to secure it in place and the tip placed on the µCT sample stage using dental wax. Imaging was performed using a CoreTOM system (TESCAN GROUP) equipped with a integrating detector and using the following parameters: X-ray tube voltage 60 kVp, X-ray tube power 15 W, integration time 350 ms, spatial resolution 0.005 mm isotropic, filter 1 mm aluminum, acquisition time ~1 h.

### Bioinformatics

Using publicly available resources from Bryant et al.^88^, we acquired the expression matrix of transcripts of interest involved in GC signaling from a range of different axolotl tissues (heart, blood vessels, skeletal muscle, gills, bone, forearm, upper arm, hand, testes, ovaries and embryo). We then calculated the average expression of each transcript across tissues and generated a heatmap with each tile color was generated based on the expression of each transcript within each tissue in relation to the average across tissues.

### Statistics

Analyses were performed using RStudio and the package glmmTMB to conduct GLMMγ. Models were created for the assayed variables, including time (when appropriate), treatment and the measured value, as well as their specified interactions. Residuals of the models were examined using the package Dharma. Individual animal ID was accounted for as a random effect in each model when measurements were paired across time. The specific contrasts were calculated from the model using package (emmeans), and correction for multiple comparisons was performed using Tukey (multiple pairwise comparisons), or Šidák’s method (comparison with baseline). In the in vivo incubation experiment, the well ID within each animal (donor animal ID) was used as a random effect to allow samples from the same animal to have different intercepts from those of other animals. In choosing the most appropriate statistical tool, GLMMγ was initially tested against linear mixed models to determine which model was a better fit for the data presented in this Article, and, throughout, GLMMγ was found to be a better or equivalent fit when examining the divergence from linearity in QQ plots as well as residuals. In the case of statistical comparisons of fold changes, the data were analyzed on the log scale for each individual replicate and then back transformed to the response scale for plotting.

### Reporting summary

Further information on research design is available in the Nature Portfolio Reporting Summary linked to this article.

## Online content

Any methods, additional references, Nature Portfolio reporting summaries, source data, extended data, supplementary information, acknowledgements, peer review information; details of author contributions and competing interests; and statements of data and code availability are available at 10.1038/s41684-026-01692-y.

## Supplementary information

Supplementary Information Supplementary Figs. 1–5 and Tables 1–3.

Reporting Summary

## Data Availability

All data are available upon reasonable request to the corresponding author. The complete dataset from the µCT is available via figshare at 10.6084/m9.figshare.30903434.v1 (ref. ^93^).
