# Supplementary material for: The adrenal stress response involves distinct dynamics of both cortisol and corticosterone in the axolotl salamander
Source: Lab Anim (NY). 2026 Mar 2;55(4):117–36. doi: 10.1038/s41684-026-01692-y (PMC13043310; doi:10.1038/s41684-026-01692-y)
Supplement: Supplementary file 1 — Supplementary Figs. 1–5 and Tables 1–3. [file 41684_2026_1692_MOESM1_ESM.pdf]

lab animal

Supplementary information

https://doi.org/10.1038/s41684-026-01692-y

The adrenal stress response involves distinct dynamics of both cortisol and corticosterone in the axolotl salamander

In the format provided by the authors and unedited

The adrenal stress response involves distinct dynamics of both cortisol and corticosterone in the axolotl salamander.

SUPPLEMENTARY INFORMATION

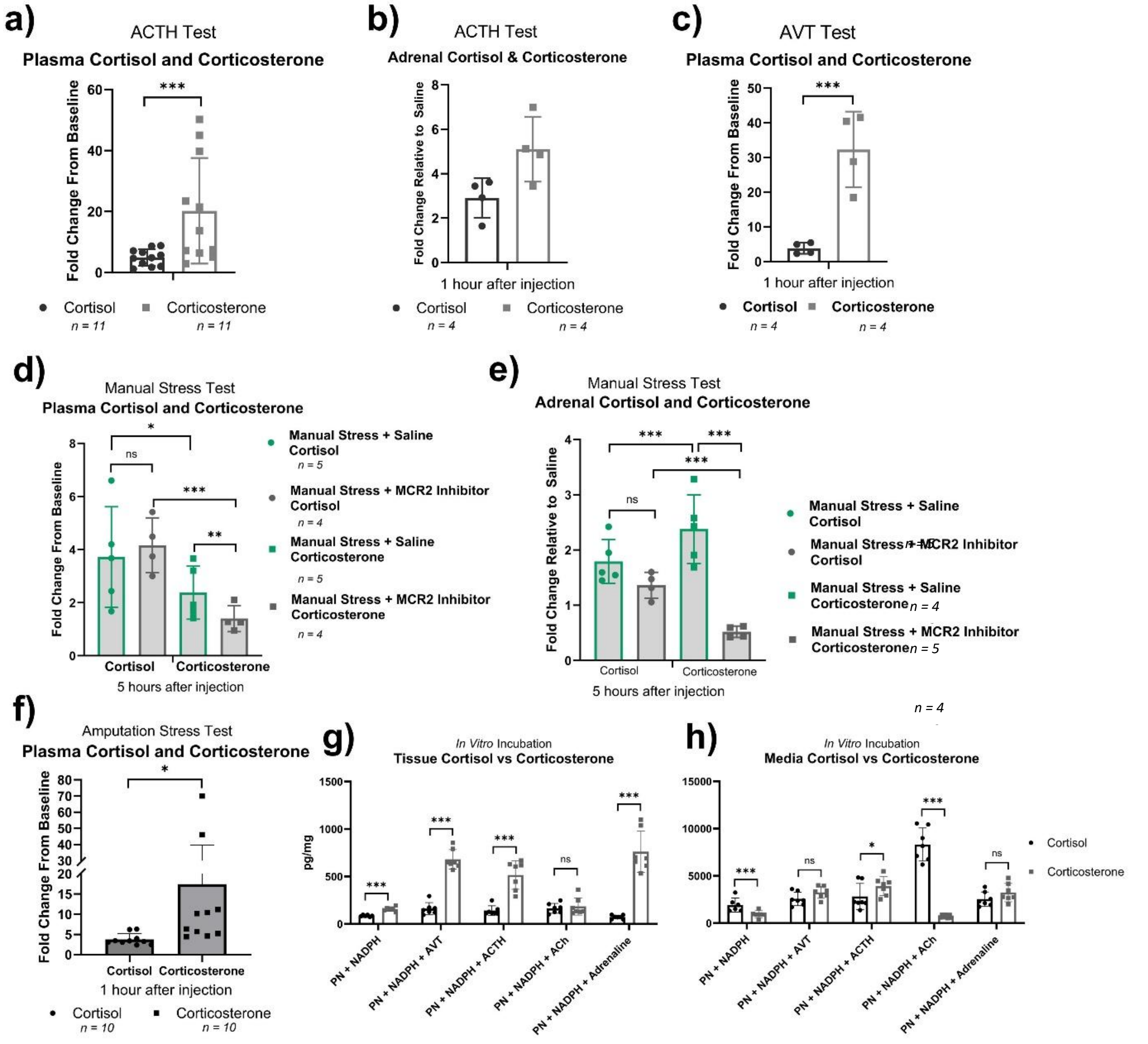

Supplementary Figure 1. Comparison of Cortisol versus Corticosterone. Statistical analysis of fold changes was performed on log transformed values of each replicate and back transformed to the response scale for plotting. a) Fold changes from baseline of plasma cortisol versus corticosterone at 1 hour after injection of ACTH (adrenocorticotropic hormone) (supplementary to Figure 2). b) Fold changes from average of saline group of adrenal tissue cortisol versus corticosterone at 1 hour after injection of ACTH (supplementary to Figure 2). c) Fold change from baseline of plasma cortisol versus corticosterone at 1 hour after injection of AVT (Arginine vasotocin) (supplementary to Figure 3). d) Fold change from baseline of plasma cortisol versus corticosterone at 5 hours after manual stress or manual stress with MCR2 (melanocortin receptor 2**)** inhibitor (supplementary to Figure 4). e) Fold change from average of saline group of adrenal tissue cortisol versus corticosterone at 5 hours after manual stress or manual stress with MCR2 inhibitor (supplementary to Figure 4). f) Fold changes from baseline of plasma cortisol versus corticosterone at 1 hour after sham of amputation surgery (supplementary to

Figure 5). g) Comparisons of tissue concentrations (proxy for synthesis) of cortisol versus corticosterone in *in vitro* samples after incubation for 2 hours with different stimulatory factors as indicated on x-axis (supplementary to Figure 6). h) Comparisons of media concentrations (proxy for release) of cortisol versus corticosterone in *in vitro* samples after incubation for 2 hours with different stimulatory factors as indicated on x-axis (supplementary to Figure 6). All error bars display standard deviation. Squares and circles in bar graphs represent individual replicates. Asterix indicates statistical significance between cortisol and corticosterone of paired samples. Statistical significance was determined by generalized linear mixed modelling with gamma distribution and Tukey post hoc tests * = p <0.05, ** = p <0.005 and *** = p <0.0001. All statistical analysis shown in Supplementary Table 2.

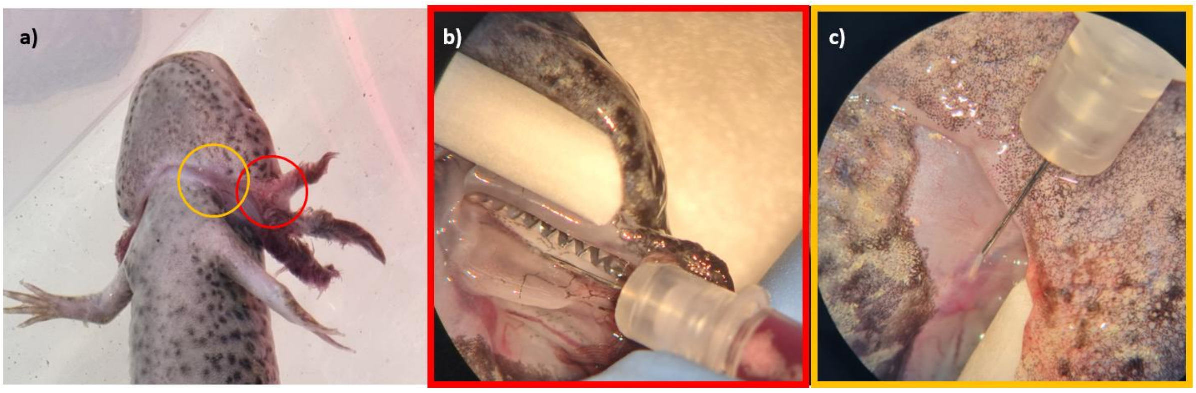

Supplementary Figure 2. Blood sampling and intravenous injection technique. a) anatomical localization of where the gill hood is gently lifted with a cotton swab to reveal the gill artery from which a blood sample is collected (b/ red circle) and the jugular vein used for intravenous injections (yellow circle/ c).

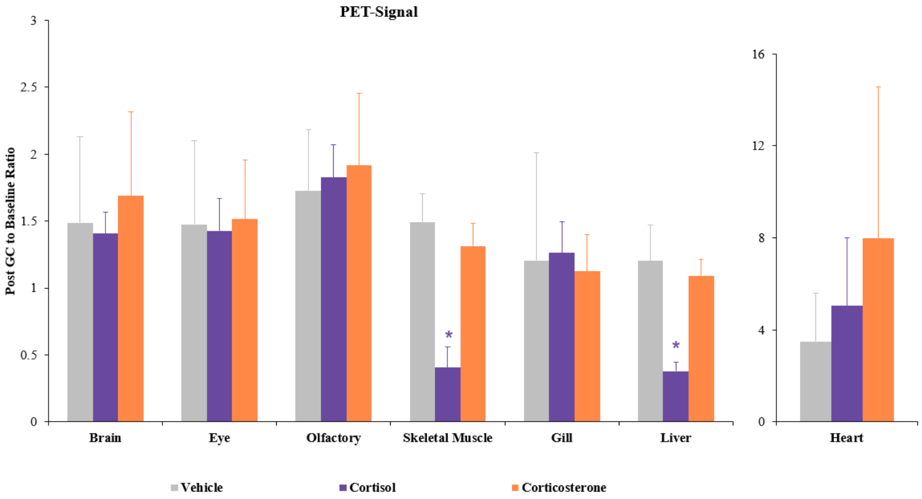

Supplementary Figure 3. PET-imaging data presented in Figure 8c including all analyzed tissues.

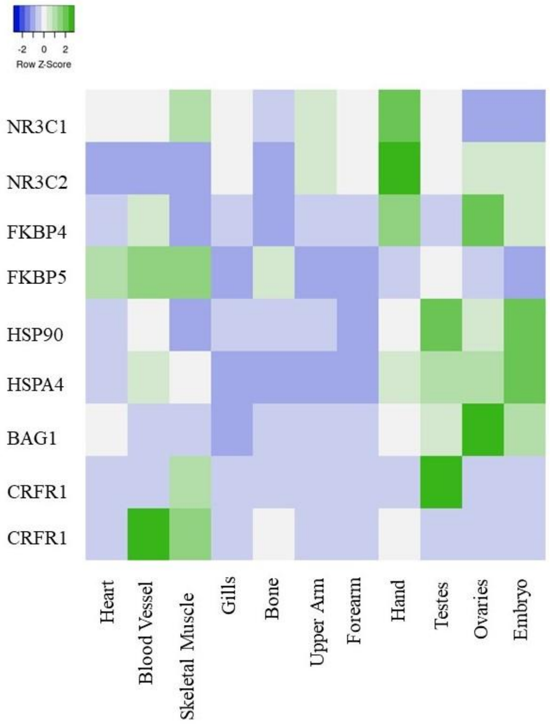

Supplementary Figure 4. Heatmap of glucocorticoid related gene transcripts. Each square show degree of up- or downregulation compared to the average transcript count of the gene across all included tissues.

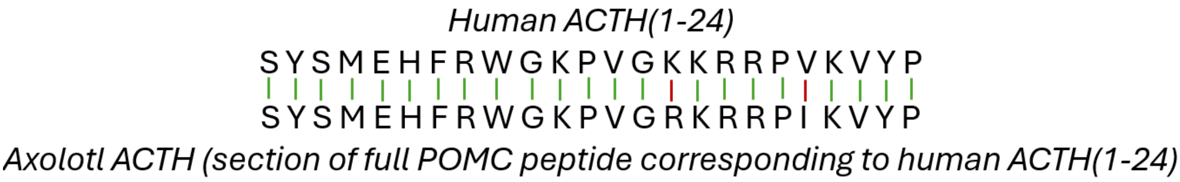

Supplementary Figure 5. Homology of human and axolotl ACTH(1-24). Alignment of the amino acid sequences of human ACTH (adrenocorticotropic hormone) (amino acids 1-24) available from NCBI (accession number CAA00890) and the corresponding region of axolotl POMC (Proopiomelanocortin) (ACTH is a cleavage product of POMC). The axolotl amino acid sequence is available via axolotl-omnics.org (sequence ID: AMEX60DD201032740.1). The sequences are 91.7 % identical with one lysine to arginine and one valine to isoleucin substitution, both of which do not convey any change in charge.

Supplementary Table 1. Glucocorticoid related genes in axolotl.

Gene name	Contig Brandt Transcriptome / Gene ID axolotl-omnics	Sequence homology to human mRNA within homologous region	Top 3 species sequence homology	Gene Function
**NR3C1 / Glucocorticoid Receptor**	c1086420_g2^sp / AMEX60DD201028813.1	Multiple human transcript variants. Transcript variant 1: 74.37%	Japanese Firebelly Newt (83.98%), Chicken (78.6 %) and Chinese alligator (77.94%)	The glucocorticoid receptor. Upon activation acts either as transcription factor by binding to glucocorticoid-response elements (GRE) or modulates other transcription factors. Affects wide range of functions including immune response, proliferation and differentiation.
**NR3C2 / Mineralocorticoid Receptor**	c1057299_g1^sp / AMEX60DD10204520.3	Multiple human transcript variants. Transcript variant 1: 71.75%	Slender lungfish (82.98 %), Mouse (77.59%) and Xenopus leavis (77.30%)	Mineralocorticoid receptor. Primarily binds to aldosterone but also glucocorticoids. Acts as transcription factor by binding to mineralocorticoid response elements (MRE), the main downstream effects of which is regulation of salt and water balance, blood pressure and potassium levels.
**FKBP4 / Peptidyl-prolyl cis-trans isomerase FKBP4**	c1079172_g1^sp / AMEX60DD301006425.1	70.16%	European fire-bellied toad (77.25%), Large yellow croaker fish (75.68%) and Kiwi bird (75.39%)	Chaperone protein that interacts with corticoid receptor complexes via HSP90. Role in intracellular trafficking of the receptors.
**FKBP5 / Peptidyl-prolyl cis-trans isomerase FKBP5**	c1060347_g1^sp / AMEX60DD201008793.4	75.10%	Two-lined caecilian (78.07%), Gaboon caecilian (77.4%) and Gavial crocodile (75.80%).	Analogous to FKBP4
**HSP90 / Heat shock protein 90**	c1063109_g1^sp / AMEX60DD201011360.1	77.72%	Iberian ribbed newt (86.74%), Asiatic toad (83.80%) and Pompano fish (83.31%)	Interacts with the glucocorticoid receptor complex. Regulates the stability, function and binding affinity of the receptor. Enhances ligand binding https://pubmed.ncbi.nlm.nih.gov/24949977/.

**HSPA4 / Heat shock protein 70 kDa protein 4**	c1061708_g2^sp / AMEX60DD201028834.2	76.02%	Chinese pond turtle (79.53%), Gaboon caecilian (79.42%) and Green sea turtle (79.39%)	Interacts with the glucocorticoid receptor complex. Regulates the stability, function and binding affinity of the receptor. Inhibits ligand affinity by unfolding the GR protein https://pubmed.ncbi.nlm.nih.gov/24949977/
**BAG1 / BAG family molecular chaperone regulator 1**	c1070289_g1^sp / AMEX60DD201038933.1	Non-homologous (>25%)	Gaboon caecilian (77.63%), Yellow-throated sandgrouse (77.07%) and Prairie chicken (76.99%)	Promotes the release of ADP from HSPA4, triggering break down of complexed in which HSPA4 is involved such as the glucocorticoid receptor complex
**CRFR1 / Corticotropin-releasing factor receptor 1**	c1077859_g4^sp / AMEX60DD201009701.1	81.02%	Two-lined caecilian (84.56%), Microcaecilia unicolor (84.29%) and Common parakeet (84.06%)	Receptor for corticotrophin releasing hormone (CRH) found in the central and peripheral nervous system. Activation increases cyclic-AMP. Mediates stress responses.
**CRFR2 / Corticotropin-releasing factor receptor 2**	c1066692_g1^sp / AMEX60DD201009701.1	74.27%	Two-lined caecilian (84.56%), Microcaecilia unicolor (84.29%) and Common parakeet (84.06%)	Receptor for corticotrophin releasing hormone (CRH) found in the central and peripheral nervous system. Proposed to have modulating effect on stress responses https://www.ncbi.nlm.nih.gov/pmc/articles/PMC6757589/

Supplementary Table 2. Significant Results from statistical analysis.

<table>
<tr><th>Fig</th><th>n number</th><th>Statistical test used,</th><th>Correction for multiple testing used</th><th>Model specification</th><th>Comparison</th><th>Contrast</th><th>ratio</th><th>SE</th><th>Z ratio</th><th>P value</th></tr>
<tr><td rowspan="8">2
b&c</td><td rowspan="8">Saline n=9,
ACTH n=11.</td><td rowspan="8">Generalized linear mixed model, gamma distribution,</td><td rowspan="8">Sidak, within each group between baseline and later time points.

Tukey between different groups</td><td rowspan="8">glmmTMB(Concentration ~ Hormone* Time *Tx+ (1|ID_anita), family = Gamma(link = "log"), data=axolotl),

effect of time from baseline: emmeans (model, specs = trt.vs.ctrl ~ Time|Tx|Hormone, type = "response", adjust = "sidak"

effect of group at each time emmeans(model, specs = pairwise ~ Tx|Time|Hormone, type = "response", adjust = "Tukey")

Significant contrasts shown in columns.</td><td rowspan="2">Tx = ACTH, Hormone = corticosterone</td><td>1 / baseline</td><td>12.448</td><td>3.301</td><td>9.507</td><td><.0001</td></tr>
<tr><td>3 / baseline</td><td>4.564</td><td>1.229</td><td>5.637</td><td><.0001</td></tr>
<tr><td rowspan="2">Tx = ACTH, Hormone = cortisol</td><td>1 / baseline</td><td>3.690</td><td>0.973</td><td>4.950</td><td><.0001</td></tr>
<tr><td>3 / baseline</td><td>1.991</td><td>0.532</td><td>2.578</td><td>0.0295</td></tr>
<tr><td>Time = 1, Hormone = corticosterone:</td><td>saline / ACTH</td><td>0.105</td><td>0.0339</td><td>-6.994</td><td><.0001</td></tr>
<tr><td>Time = 3, Hormone = corticosterone:</td><td>saline / ACTH</td><td>0.252</td><td>0.0817</td><td>-4.251</td><td><.0001</td></tr>
<tr><td>Time = 1, Hormone = cortisol</td><td>saline / ACTH</td><td>0.152</td><td>0.0488</td><td>-5.877</td><td><.0001</td></tr>
<tr><td>Time = 3, Hormone = cortisol</td><td>saline / ACTH</td><td>0.379</td><td>0.1219</td><td>-3.017</td><td>0.0026</td></tr>
<tr><td rowspan="10">2d&e</td><td rowspan="10">Saline n=3,
ACTH n=5</td><td rowspan="10">Generalized linear mixed model, gamma distribution,</td><td rowspan="10">Sidak, within each group between baseline and later time points.

Tukey between different groups</td><td rowspan="10">glmmTMB(Concentration ~ Hormone* Time *Tx+ (1|ID_anita), family = Gamma(link = "log"), data=axolotl),

effect of time from baseline: emmeans (model, specs = trt.vs.ctrl ~ Time|Tx|Hormone, type = "response", adjust = "sidak"

effect of group at each time emmeans(model, specs = pairwise ~ Tx|Time|Hormone, type = "response", adjust = "Tukey")

Significant contrasts shown in columns.</td><td>Tx = ACTH, Hormone = corticosterone:</td><td>3 / Baseline</td><td>2.011</td><td>0.5464</td><td>2.573</td><td>0.0398</td></tr>
<tr><td>Tx = saline, Hormone = corticosterone:</td><td>24 / Baseline</td><td>0.407</td><td>0.1430</td><td>-2.558</td><td>0.0415</td></tr>
<tr><td rowspan="4">Tx = ACTH, Hormone = cortisol:</td><td>1 / Baseline</td><td>0.367</td><td>0.1001</td><td>-3.677</td><td>0.0009</td></tr>
<tr><td>3 / Baseline</td><td>0.284</td><td>0.0775</td><td>-4.613</td><td><.0001</td></tr>
<tr><td>24 / Baseline</td><td>0.266</td><td>0.0726</td><td>-4.856</td><td><.0001</td></tr>
<tr><td>48 / Baseline</td><td>0.397</td><td>0.1081</td><td>-3.394</td><td>0.0028</td></tr>
<tr><td>Time = 3, Hormone = corticosterone</td><td>ACTH / saline</td><td>3.942</td><td>1.380</td><td>3.920</td><td>0.0001</td></tr>
<tr><td>Time = 24, Hormone = corticosterone</td><td>ACTH / saline</td><td>4.390</td><td>1.529</td><td>4.247</td><td><.0001</td></tr>
<tr><td>Time = 48, Hormone = corticosterone</td><td>ACTH / saline</td><td>2.024</td><td>0.707</td><td>2.019</td><td>0.0434</td></tr>
<tr><td>Time = 24, Hormone = cortisol</td><td>ACTH / saline</td><td>0.506</td><td>0.176</td><td>-1.960</td><td>0.0500</td></tr>
<tr><td rowspan="2">2fg</td><td rowspan="2">Saline n=5,
ACTH n=5</td><td rowspan="2">Generalized linear mixed model, gamma distribution,</td><td rowspan="2">Tukey between different groups</td><td rowspan="2">glmmTMB(Concentration ~ Hormone *Tx+ (1|ID_anita), family = Gamma(link = "log"), data=axolotl)

emmeans(model, specs = trt.vs.ctrl ~ Tx+ Tx|Hormone, type = "response", adjust = "Tukey")</td><td>Hormone = Corticosterone</td><td>saline / ACTH</td><td>0.198</td><td>0.0394</td><td>-8.130</td><td><.0001</td></tr>
<tr><td>Hormone = Cortisol</td><td>saline / ACTH</td><td>0.341</td><td>0.0681</td><td>-5.389</td><td><.0001</td></tr>
<tr><td rowspan="2">2hi</td><td rowspan="2">Saline n=5,
ACTH n=5</td><td rowspan="2">Generalized linear mixed model, gamma distribution,</td><td rowspan="2">Tukey between different groups</td><td rowspan="2">glmmTMB(Concentration ~ Tissue *Tx+ (1|ID_anita), family = Gamma(link = "log"), data=axolotl)

emmeans(model_1_total, specs = trt.vs.ctrl ~ Tx+ Tx|Tissue, type = "response", adjust = "Tukey")</td><td>Tissue = Adrenal</td><td>saline / ACTH</td><td>0.639</td><td>0.0929</td><td>-3.080</td><td>0.0021</td></tr>
<tr><td>Tissue = Brain</td><td>saline / ACTH</td><td>1.462</td><td>0.2126</td><td>2.610</td><td>0.0091</td></tr>
<tr><td rowspan="4">3b</td><td rowspan="4">Saline n=6,
AVT n=6</td><td rowspan="4">Generalized linear mixed model, gamma distribution,</td><td rowspan="4">Sidak, within each group between baseline and later time points.

Tukey between different groups</td><td rowspan="4">glmmTMB(Concentration ~ Time *Tx+ (1|ID_anita), family = Gamma(link = "log"), data=axolotl)
emmeans(model_1_total, specs = trt.vs.ctrl ~ Time|Tx, type = "response", adjust = "sidak")
emmeans(model, specs = pairwise ~ Tx|Time, type = "response", adjust = "Tukey")</td><td>Tx = AVT</td><td>10 min / Baseline</td><td>10.243</td><td>1.339</td><td>17.800</td><td><.0001</td></tr>
<tr><td>Tx = AVT</td><td>1 hour / Baseline</td><td>3.210</td><td>0.420</td><td>8.907</td><td><.0001</td></tr>
<tr><td>Time = 10 min:</td><td>AVT / Saline</td><td>11.115</td><td>1.461</td><td>18.321</td><td><.0001</td></tr>
<tr><td>Time = 1 hour:</td><td>AVT / Saline</td><td>2.819</td><td>0.372</td><td>7.863</td><td><.0001</td></tr>
<tr><td rowspan="2">3c,d</td><td rowspan="2">Saline n=5,
AVT n=6</td><td rowspan="2">Generalized linear mixed model, gamma distribution,</td><td rowspan="2">Tukey between different groups</td><td rowspan="2">glmmTMB(Concentration ~ Time *Tx+ (1|ID_anita), family = Gamma(link = "log"), data=axolotl)

emmeans(model, specs = trt.vs.ctrl ~ Tx|Tissue, type = "response", adjust = "Tukey")</td><td>Tissue = adrenal:</td><td>saline / AVT</td><td>0.551</td><td>0.0667</td><td>-4.922</td><td><.0001</td></tr>
<tr><td>Tissue = brain:</td><td>saline / AVT</td><td>0.117</td><td>0.0141</td><td>-17.794</td><td><.0001</td></tr>
</table>

<table>
<tr><th></th><th></th><th></th><th></th><th></th><th></th><th></th><th></th><th></th><th></th><th></th></tr>
<tr><td>3e,f</td><td>Saline n=4,
AVT n=4</td><td>Generalized linear mixed model, gamma distribution,</td><td>Sidak, within each group between baseline and later time points.

Tukey between different groups</td><td>glmmTMB(Concentration ~ Hormone* Time *Tx+ (1|ID_anita), family = Gamma(link = "log"), data=axolotl)
emmeans(model_1_total, specs = trt.vs.ctrl ~ Time|Tx|Hormone, type = "response", adjust = "sidak")</td><td>Tx = AVT, Hormone = Corticosterone</td><td>10 mins / Baseline</td><td>2.484</td><td>0.470</td><td>4.807</td><td><.0001</td></tr>
<tr><td></td><td></td><td></td><td></td><td></td><td></td><td>1 hour / Baseline</td><td>30.789</td><td>5.818</td><td>18.136</td><td><.0001</td></tr>
<tr><td></td><td></td><td></td><td></td><td></td><td></td><td>3 hours / Baseline</td><td>5.363</td><td>1.011</td><td>8.908</td><td><.0001</td></tr>
<tr><td></td><td></td><td></td><td></td><td></td><td></td><td>24 hours / Baseline</td><td>1.694</td><td>0.319</td><td>2.795</td><td>0.0206</td></tr>
<tr><td></td><td></td><td></td><td></td><td></td><td>Tx = saline, Hormone = Corticosterone</td><td>24 hours / Baseline</td><td>1.637</td><td>0.309</td><td>2.617</td><td>0.0350</td></tr>
<tr><td></td><td></td><td></td><td></td><td></td><td>Tx = AVT, Hormone = Cortisol</td><td>10 mins / Baseline</td><td>2.192</td><td>0.413</td><td>4.164</td><td>0.0001</td></tr>
<tr><td></td><td></td><td></td><td></td><td></td><td></td><td>1 hour / Baseline</td><td>3.717</td><td>0.702</td><td>6.951</td><td><.0001</td></tr>
<tr><td></td><td></td><td></td><td></td><td>contrastTx_tx_total=emmeans::emmeans(model_1_total, specs = pairwise ~ Tx|Time|Hormone, type = "response", adjust = "Tukey")</td><td>Time = 10min, Hormone = Corticosterone</td><td>AVT / saline</td><td>3.288</td><td>0.624</td><td>6.273</td><td><.0001</td></tr>
<tr><td></td><td></td><td></td><td></td><td></td><td>Time = 1 hour, Hormone = Corticosterone</td><td>AVT / saline</td><td>31.716</td><td>6.014</td><td>18.229</td><td><.0001</td></tr>
<tr><td></td><td></td><td></td><td></td><td></td><td>Time = 3 hours, Hormone = Corticosterone:</td><td>AVT / saline</td><td>5.710</td><td>1.079</td><td>9.225</td><td><.0001</td></tr>
<tr><td></td><td></td><td></td><td></td><td></td><td>Time = 24 hours, Hormone = Corticosterone:</td><td>AVT / saline</td><td>1.542</td><td>0.291</td><td>2.293</td><td>0.0218</td></tr>
<tr><td></td><td></td><td></td><td></td><td></td><td>Time = 10 mins, Hormone = Cortisol</td><td>AVT / saline</td><td>2.000</td><td>0.377</td><td>3.676</td><td>0.0002</td></tr>
<tr><td></td><td></td><td></td><td></td><td></td><td>Time = 1 hour, Hormone = Cortisol</td><td>AVT / saline</td><td>2.935</td><td>0.557</td><td>5.676</td><td><.0001</td></tr>
<tr><td>3i</td><td>Saline n=10,
AVT n=10</td><td>Generalized linear mixed model, gamma distribution,</td><td>Tukey between different groups</td><td>glmmTMB(Hct~Tx, family = Gamma(link = "log"), data=axolotl)

emmeans(model_1_total, specs = trt.vs.ctrl ~ Tx, type = "response", adjust = "Tukey")</td><td></td><td>saline / AVT</td><td>2.57</td><td>0.446</td><td>5.444</td><td><.0001</td></tr>
<tr><td>3g,h</td><td>Saline n=5, AVT n=6</td><td>Generalized linear mixed model, gamma distribution,</td><td>Tukey between different groups</td><td>glmmTMB(Concentration ~ Tx* Hormone + (1|ID_anita), family = Gamma(link = "log"), data=axolotl)

emmeans(model_1_total, specs = trt.vs.ctrl ~ Tx|Hormone, type = "response", adjust = "Tukey")</td><td>Hormone = corticosterone</td><td>saline / AVT</td><td>0.290</td><td>0.0449</td><td>-7.999</td><td><.0001</td></tr>
<tr><td>4b</td><td>Manual stress n=5,
Manual stress + MCR2 inhibitor n=5</td><td>Generalized linear mixed model, gamma distribution,</td><td>Sidak, within each group between baseline and later time points.

Tukey between different groups</td><td>glmmTMB(Concentration ~ Tx *Time+ (1|ID_anita), family = Gamma(link = "log"), data=axolotl)
emmeans(model_1_total, specs = trt.vs.ctrl ~ Time+ Time|Tx, type = "response", adjust = "sidak")</td><td>Tx = MS</td><td>3 hours / Baseline</td><td>1.599</td><td>0.1347</td><td>5.567</td><td><.0001</td></tr>
<tr><td></td><td></td><td></td><td></td><td></td><td></td><td>5 hours / Baseline</td><td>1.254</td><td>0.1057</td><td>2.683</td><td>0.0289</td></tr>
<tr><td></td><td></td><td></td><td></td><td></td><td>Tx = MS_MCRi</td><td>1 hour / Baseline</td><td>0.799</td><td>0.0674</td><td>-2.660</td><td>0.0309</td></tr>
<tr><td></td><td></td><td></td><td></td><td></td><td></td><td>3 hours / Baseline</td><td>1.394</td><td>0.1176</td><td>3.942</td><td>0.0003</td></tr>
<tr><td></td><td></td><td></td><td></td><td></td><td></td><td>5 hours / Baseline</td><td>0.759</td><td>0.0640</td><td>-3.266</td><td>0.0043</td></tr>
<tr><td></td><td></td><td></td><td></td><td></td><td></td><td>24 hours / Baseline</td><td>0.751</td><td>0.0633</td><td>3.395</td><td>0.0027</td></tr>
<tr><td></td><td></td><td></td><td></td><td>emmeans(model_1_total, specs = trt.vs.ctrl ~ Tx|Time, type = "response", adjust = "Tukey")</td><td>Time = 5 hours:</td><td>MS_MCRi / MS</td><td>0.696</td><td>0.0642</td><td>-3.935</td><td>0.0001</td></tr>
<tr><td>4c,d</td><td>Saline n=4
Manual stress n=5,
Manual stress + MCR2 inhibitor n=4</td><td>Generalized linear mixed model, gamma distribution,</td><td>Tukey between different groups</td><td>glmmTMB(Concentration ~ Tx* Tissue + (1|ID_anita), family = Gamma(link = "log"), data=axolotl)
emmeans::emmeans(model_1_total, specs = pairwise ~ Tx|Tissue, type = "response", adjust = "Tukey")</td><td>Tissue = Adrenal</td><td>Saline / MS</td><td>1.581</td><td>0.281</td><td>2.577</td><td>0.0269</td></tr>
<tr><td>4e,f</td><td>Manual stress n=5,
Manual stress + MCR2 inhibitor n=4</td><td>Generalized linear mixed model, gamma distribution,</td><td>Sidak, within each group between baseline and later time points.

Tukey between different groups</td><td>glmmTMB(Concentration ~ Hormone* Time *Tx+ (1|ID_anita), family = Gamma(link = "log"), data=axolotl)

emmeans(model_1_total, specs = trt.vs.ctrl ~ Time|Tx|Hormone, type = "response", adjust = "sidak")</td><td>Tx = MS, Hormone = Corticosterone:</td><td>3 hours / Baseline</td><td>1.704</td><td>0.280</td><td>3.237</td><td>0.0048</td></tr>
<tr><td></td><td></td><td></td><td></td><td></td><td></td><td>5 hours / Baseline</td><td>2.240</td><td>0.369</td><td>4.902</td><td><.0001</td></tr>
<tr><td></td><td></td><td></td><td></td><td></td><td>Tx = MS, Hormone = Cortisol:</td><td>5 hours / Baseline</td><td>3.521</td><td>0.579</td><td>7.649</td><td><.0001</td></tr>
<tr><td></td><td></td><td></td><td></td><td></td><td>Tx = MS_MCRi, Hormone = Cortisol:</td><td>5 hours / Baseline</td><td>4.113</td><td>0.757</td><td>7.687</td><td><.0001</td></tr>
<tr><td></td><td></td><td></td><td></td><td>emmeans(model_1_total, specs = pairwise ~ Tx|Time|Hormone,</td><td></td><td>MS / MS_MCRi</td><td>1.637</td><td>0.286</td><td>2.823</td><td>0.0048</td></tr>
</table>

<table>
<tr><td></td><td></td><td></td><td></td><td>type = "response", adjust = "Tukey")</td><td>Time = 3 hours, Hormone = Corticosterone:
Time = 5 hours, Hormone = Corticosterone:</td><td>MS / MS_MCRi</td><td>1.884</td><td>0.329</td><td>3.631</td><td>0.0003</td></tr>
<tr><td>4g,h</td><td>Manual stress n=5,
Manual stress + MCR2 inhibitor n=4</td><td>Generalized linear mixed model, gamma distribution,</td><td>Tukey between different groups</td><td>glmmTMB(Concentration ~ Tx* Hormone + (1|ID_anita), family = Gamma(link = "log"), data=axolotl)

emmeans(model_1_total, specs = pairwise ~ Tx|Hormone, type = "response", adjust = "Tukey")</td><td>Hormone = Cortisol:</td><td>Saline / MS</td><td>0.542</td><td>0.0830</td><td>-4.001</td><td>0.0002</td></tr>
<tr><td></td><td></td><td></td><td></td><td></td><td>Hormone = Corticosterone:</td><td>Saline / MS</td><td>0.417</td><td>0.0636</td><td>-5.737</td><td><.0001</td></tr>
<tr><td></td><td></td><td></td><td></td><td></td><td></td><td>Saline / MS_MCRi</td><td>1.908</td><td>0.3078</td><td>4.007</td><td>0.0002</td></tr>
<tr><td></td><td></td><td></td><td></td><td></td><td></td><td>MS / MS_MCRi</td><td>4.571</td><td>0.7376</td><td>9.420</td><td><.0001</td></tr>
<tr><td>4j,k,l</td><td>Manual stress: n=4</td><td>Generalized linear mixed model, gamma distribution,</td><td>Sidak, within each group between baseline and later time points.</td><td>glmmTMB(Concentration ~ Hormone *Time+ (1|ID_anita), family = Gamma(link = "log"), data=axolotl)</td><td>Hormone = adrenaline</td><td>10 min / Baseline</td><td>27.429</td><td>14.429</td><td>6.295</td><td><.0001</td></tr>
<tr><td></td><td></td><td></td><td></td><td></td><td>Hormone = Dopamine:</td><td>10 min / Baseline</td><td>30.031</td><td>15.870</td><td>6.438</td><td><.0001</td></tr>
<tr><td></td><td></td><td></td><td></td><td></td><td></td><td>3 hours / Baseline</td><td>0.223</td><td>0.117</td><td>-2.857</td><td>0.0212</td></tr>
<tr><td>5b,c</td><td>Sham n=10, Amputation n=10</td><td>Generalized linear mixed model, gamma distribution,</td><td>Sidak, within each group between baseline and later time points.

Tukey between different groups</td><td>glmmTMB(Concentration ~ Hormone* Time *Tx+ (1|ID_anita), family = Gamma(link = "log"), data=axolotl
emmeans(model_1_total, specs = trt.vs.ctrl ~ Time|Tx|Hormone, type = "response", adjust = sidak")

emmeans(model_1_total, specs = pairwise ~ Tx|Time|Hormone, type = "response", adjust = "Tukey")
contrastTx_tx_total</td><td>Tx = amputation, Hormone = corticosterone</td><td>10 min / Baseline</td><td>4.841</td><td>1.191</td><td>6.413</td><td><.0001</td></tr>
<tr><td></td><td></td><td></td><td></td><td></td><td></td><td>1 hour / Baseline</td><td>10.618</td><td>2.638</td><td>9.509</td><td><.0001</td></tr>
<tr><td></td><td></td><td></td><td></td><td></td><td>Tx = sham, Hormone = corticosterone:</td><td>10 min / Baseline</td><td>2.017</td><td>0.499</td><td>2.834</td><td>0.0228</td></tr>
<tr><td></td><td></td><td></td><td></td><td></td><td></td><td>1 hour / Baseline</td><td>3.243</td><td>0.802</td><td>4.760</td><td><.0001</td></tr>
<tr><td></td><td></td><td></td><td></td><td></td><td></td><td>96 hours / Baseline</td><td>1.981</td><td>0.487</td><td>2.779</td><td>0.0269</td></tr>
<tr><td></td><td></td><td></td><td></td><td></td><td>Tx = amputation, Hormone = cortisol:</td><td>1 hour / Baseline</td><td>3.655</td><td>0.897</td><td>5.278</td><td><.0001</td></tr>
<tr><td></td><td></td><td></td><td></td><td></td><td>Tx = sham, Hormone = cortisol:</td><td>1 hour / Baseline</td><td>1.897</td><td>0.467</td><td>2.599</td><td>0.0458</td></tr>
<tr><td></td><td></td><td></td><td></td><td></td><td>Tx = 1 hour, Hormone = cortisol</td><td>Amputation / sham</td><td>2.148</td><td>0.543</td><td>3.022</td><td>0.0025</td></tr>
<tr><td></td><td></td><td></td><td></td><td></td><td>Tx = 10 min, Hormone = corticosterone</td><td>Amputation / sham</td><td>2.533</td><td>0.642</td><td>3.667</td><td>0.0002</td></tr>
<tr><td></td><td></td><td></td><td></td><td></td><td>Tx = 1 hour, Hormone = corticosterone</td><td>Amputation / sham</td><td>3.455</td><td>0.879</td><td>4.873</td><td>< .0001</td></tr>
<tr><td>5d</td><td>Sham n=10, Amputation n=10</td><td>Generalized linear mixed model, gamma distribution,</td><td>Sidak, within each group between baseline and later time points.

Tukey between different groups</td><td>model_1_total=glmmTMB(BG ~Time *Tx+ (1|ID_anita), family = Gamma(link = "log"), data=axolotl)
emmeans(model_1_total, specs = trt.vs.ctrl ~ Time|Tx, type = "response", adjust = "sidak")
contrastTx_time_total_1</td><td>Tx = amputation</td><td>24 hours / Baseline Inf</td><td>0.874</td><td>0.0434</td><td>-2.709</td><td>0.0333</td></tr>
<tr><td></td><td></td><td></td><td></td><td></td><td>Tx = sham</td><td>5 hours / Baseline</td><td>0.868</td><td>0.0431</td><td>-2.851</td><td>0.0216</td></tr>
<tr><td></td><td></td><td></td><td></td><td></td><td></td><td>24 hours / Baseline</td><td>0.863</td><td>0.0429</td><td>-2.970</td><td>0.0148</td></tr>
<tr><td>5 e</td><td>Sham n=10, Amputation n=10</td><td>Generalized linear mixed model, gamma distribution,</td><td>Sidak, within each group between baseline and later time points.

Tukey between different groups</td><td>model_1_total=glmmTMB(BG ~Time *Tx+ (1|ID_anita), family = Gamma(link = "log"), data=axolotl)
emmeans(model_1_total, specs = trt.vs.ctrl ~ Time|Tx, type = "response", adjust = "sidak")
contrastTx_time_total_1

emmeans(model_1_total, specs = pairwise ~ Tx|Time, type = "response", adjust = "Tukey")</td><td>Tx = amputation:</td><td>10 min / Baseline</td><td>1.378</td><td>0.1035</td><td>4.265</td><td>0.0001</td></tr>
<tr><td></td><td></td><td></td><td></td><td></td><td></td><td>1 hour / Baseline</td><td>2.249</td><td>0.1691</td><td>10.780</td><td><.0001</td></tr>
<tr><td></td><td></td><td></td><td></td><td></td><td></td><td>5 hours / Baseline</td><td>2.293</td><td>0.1725</td><td>11.036</td><td><.0001</td></tr>
<tr><td></td><td></td><td></td><td></td><td></td><td>Tx = sham:</td><td>10 min / Baseline</td><td>1.276</td><td>0.0960</td><td>3.247</td><td>0.0058</td></tr>
<tr><td></td><td></td><td></td><td></td><td></td><td></td><td>1 hour / Baseline</td><td>2.152</td><td>0.1622</td><td>10.168</td><td><.0001</td></tr>
<tr><td></td><td></td><td></td><td></td><td></td><td></td><td>5 hours / Baseline</td><td>2.349</td><td>0.1770</td><td>11.335</td><td><.0001</td></tr>
<tr><td></td><td></td><td></td><td></td><td></td><td>Time = Baseline</td><td>amputation / sham</td><td>0.789</td><td>0.0931</td><td>-2.011</td><td>0.0444</td></tr>
<tr><td></td><td></td><td></td><td></td><td></td><td>Time = 5 hours</td><td>amputation / sham</td><td>0.770</td><td>0.0909</td><td>-2.213</td><td>0.0269</td></tr>
<tr><td></td><td></td><td></td><td></td><td></td><td>Time = 24 hours</td><td>amputation / sham</td><td>0.734</td><td>0.0866</td><td>-2.623</td><td>0.0087</td></tr>
<tr><td>6 c, e</td><td>Adrenal issue taken from n=10 axolotls, each animals adrenal tissue divided into 8 sections, and paired so 2 pieces per well. n=6 for negative and positive controls and n=7 wells per treatment. Tissue measurements</td><td>Generalized linear mixed model, gamma distribution,</td><td>Tukey between different treatments.</td><td>model_1_total=glmmTMB(Tissue ~ Tx * Output+ (1|ID_anita:Animal), family = Gamma(link = "log"), data=axolotl)

emmeans(model_1_total, specs = trt.vs.ctrl ~ Tx+ Tx|Output, type = "response", adjust = "sidak")</td><td>Output = Cortisol</td><td>Positive control / Negative Control</td><td>12.2065</td><td>1.82587</td><td>16.726</td><td><.0001</td></tr>
<tr><td></td><td></td><td></td><td></td><td></td><td></td><td>Positive control / Syn</td><td>0.6255</td><td>0.09021</td><td>- 3.253</td><td>0.0145</td></tr>
<tr><td></td><td></td><td></td><td></td><td></td><td></td><td>Positive control / Ach</td><td>0.5353</td><td>0.07723</td><td>-4.332</td><td>0.0002</td></tr>
<tr><td></td><td></td><td></td><td></td><td></td><td></td><td>Positive control / AVT</td><td>0.5527</td><td>0.07982</td><td>-4.106</td><td>0.0006</td></tr>
<tr><td></td><td></td><td></td><td></td><td></td><td></td><td>Negative Control / Syn</td><td>0.0512</td><td>0.00740</td><td>-20.586</td><td><.0001</td></tr>
<tr><td></td><td></td><td></td><td></td><td></td><td></td><td>Negative Control / Epi</td><td>0.0974</td><td>0.01405</td><td>-16.139</td><td><.0001</td></tr>
<tr><td></td><td></td><td></td><td></td><td></td><td></td><td>Negative Control / Ach</td><td>0.0439</td><td>0.00633</td><td>-21.655</td><td><.0001</td></tr>
<tr><td></td><td></td><td></td><td></td><td></td><td></td><td>Negative Control / AVT</td><td>0.0453</td><td>0.00654</td><td>-21.420</td><td><.0001</td></tr>
<tr><td></td><td></td><td></td><td></td><td></td><td></td><td>Syn / Epi</td><td>1.9000</td><td>0.26359</td><td>4.626</td><td>0.0001</td></tr>
</table>

						Epi / Ach	0.4504	0.06252	-5.746	<.0001
						Epi / AVT	0.4650	0.06459	-5.512	<.0001
					Output = Corticosterone:	Positive Control/ Negative Control	2.5251	0.37759	6.194	<.0001
						Positive Control / Syn	0.3120	0.04501	-8.074	<.0001
						Positive Control / Epi	0.2095	0.03021	-10.838	<.0001
						Positive Control / AVT	0.2326	0.03350	-10.126	<.0001
						Negative Control / Syn	0.1235	0.01783	-14.488	<.0001
						Negative Control / Epi	0.0830	0.01197	-17.253	<.0001
						Negative Control / Ach	0.3518	0.05091	-7.219	<.0001
						Negative Control / AVT	0.0921	0.01327	-16.550	<.0001
						Syn / Epi	0.6716	0.09319	-2.869	0.0473
						Syn / Ach	2.8476	0.39594	7.526	<.0001
						Epi / Ach	4.2402	0.58985	10.385	<.0001
						Ach / AVT	0.2618	0.03641	-9.636	<.0001
6 d, f		Adrenal issue taken from n=10 axolotls, each animals adrenal tissue divided into 8 sections, and paired so 2 pieces per well. n=6 for negative and positive controls and n=7 wells per treatment. Media measurements	Generalized linear mixed model, gamma distribution,	Tukey between different treatments.	model_1_total=glmmTMB(Tissue ~ Tx * Output+ (1|ID_anita:Animal), family = Gamma(link = "log"), data=axolotl) emmeans(model_1_total, specs = pairwise ~ Tx+ Tx|Output, type = "response", adjust = "Tukey")	Output = Cortisol	Positive Control / Negative Control	97.31935	1.67e+01	26.733	<.0001
						Positive Control / Ach	0.22632	3.72e-02	-9.047	<.0001
						Negative Control / Syn	0.00703	1.17e-03	-29.856	<.0001
						Negative Control / Epi	0.00763	1.26e-03	-29.629	<.0001
						Negative Control / Ach	0.00233	3.82e-04	-36.907	<.0001
						Negative Control / AVT	0.00757	1.24e-03	-29.679	<.0001
						Syn / Ach	0.33097	5.25e-02	-6.972	<.0001
						Epi / Ach	0.30473	4.80e-02	-7.540	<.0001
						Ach / AVT	3.25311	5.12e-01	7.488	<.0001
					Output = Corticosterone:	Positive Control / Negative Control	3.10702	5.32e-01	6.625	<.0001
						Positive Control / Syn	0.23767	3.91e-02	-8.726	<.0001
						Positive Control / Epi	0.28940	4.76e-02	-7.538	<.0001
						Positive Control / AVT	0.28663	4.71e-02	-7.601	<.0001
						Negative Control / Syn	0.07650	1.26e-02	-15.567	<.0001
						Negative Control / Epi	0.09314	1.54e-02	-14.386	<.0001
						Negative Control / Ach	0.41958	6.92e-02	-5.268	<.0001
						Negative Control / AVT	0.09225	1.52e-02	-14.455	<.0001
						Syn / Ach	5.48509	8.64e-01	10.800	<.0001
						Epi / Ach	4.50464	7.10e-01	9.548	<.0001
						Ach / AVT	0.21987	3.46e-02	-9.622	<.0001
8 c		Vehicle n=4, Cortisol n=4, Corticosterone n=4	Generalized linear mixed model, gamma distribution,	Tukey between different treatments.	glmmTMB(Relative_Signal_Intensity ~ Tx *Tissue+ (1|ID_anita), family = Gamma(link = "log"), data=axolotl) emmeans(model_1_total, specs = trt.vs.ctrl ~ Tx +Tx|Tissue, type = "response", adjust = "Tukey")	Tissue = Skeletal Muscle	Cortisol / Vehicle	0.266	0.0681	-5.172	<.0001
					Tissue = Liver	Cortisol / Vehicle	0.316	0.0808	-4.505	<.0001
					Tissue = Heart	Corticosterone / Vehicle	2.184		2.998	0.0054
8 e		Saline n=3, ACTH n=4	Generalized linear mixed model, gamma distribution,	Sidak between baseline and later timepoints, tukey between different treatments.	model_1_total=glmmTMB(Relative_BG ~ Tx *Time+ (1|ID_anita), family = Gamma(link = "log"), data=axolotl) emmeans(model_1_total, specs = trt.vs.cntl ~ Time|Tx, type = "response", adjust = "sidak")	Tx = ACTH	1 hour / Baseline	1.22	0.0663	3.696	0.0009
						3 hours / Baseline	1.24	0.0673	3.968	0.0003
						5 hours / Baseline	1.27	0.0688	4.362	0.0001
					emmeans(model_1_total, specs = trt.vs.ctrl ~ Tx +Tx|Time, type = "response", adjust = "Tukey")	Time = 5 hours	ACTH / Saline	1.211	0.0991	2.337	0.0194
8 f		Vehicle n=6, Cortisol n=6,		Sidak, within each group between	model_1_fold=glmmTMB(Fold_Change~Tx*Time+	Tx = DMSO (Vehicle)	3 hours / Baseline	1.73	0.259	3.676	0.0009

<table>
<tr><td></td><td>Corticosterone n=6</td><td></td><td>baseline and later time points, and between treatment and control at each time point.</td><td>(1|ID_anita), family = Gamma(link = "log"), data=grouped_df) emmeans(model_1_fold, specs = trt.vs.ctrl ~ Time|Tx, type = "response", adjust = "sidak")</td><td></td><td>24 hours / Baseline</td><td>3.48</td><td>0.521</td><td>8.328</td><td><.0001</td></tr>
<tr><td></td><td></td><td></td><td></td><td></td><td>Tx = Corticosterone</td><td>2 hours / Baseline</td><td>1.49</td><td>0.222</td><td>2.670</td><td>0.0300</td></tr>
<tr><td></td><td></td><td></td><td></td><td></td><td></td><td>3 hours / Baseline</td><td>1.90</td><td>0.284</td><td>4.311</td><td>0.0001</td></tr>
<tr><td></td><td></td><td></td><td></td><td></td><td></td><td>24 hours / Baseline</td><td>3.97</td><td>0.594</td><td>9.234</td><td><.0001</td></tr>
<tr><td></td><td></td><td></td><td></td><td></td><td>Tx = Cortisol</td><td>1 hour / Baseline</td><td>1.90</td><td>0.284</td><td>4.285</td><td>0.0001</td></tr>
<tr><td></td><td></td><td></td><td></td><td></td><td></td><td>2 hours / Baseline</td><td>2.26</td><td>0.337</td><td>5.447</td><td><.0001</td></tr>
<tr><td></td><td></td><td></td><td></td><td></td><td></td><td>3 hours / Baseline</td><td>2.52</td><td>0.376</td><td>6.183</td><td><.0001</td></tr>
<tr><td></td><td></td><td></td><td></td><td></td><td></td><td>24 hours / Baseline</td><td>4.45</td><td>0.665</td><td>9.987</td><td><.0001</td></tr>
<tr><td></td><td></td><td></td><td></td><td>emmeans(model_1_fold, specs = trt.vs.ctrl ~Tx|Time, type = "response", adjust = "sidak")</td><td>Time = 1 h</td><td>Cortisol / DMSO</td><td>1.50</td><td>0.235</td><td>2.608</td><td>0.0181</td></tr>
<tr><td></td><td></td><td></td><td></td><td></td><td>Time = 2 h</td><td>Cortisol / DMSO</td><td>1.48</td><td>0.231</td><td>2.456</td><td>0.0088</td></tr>
<tr><td></td><td></td><td></td><td></td><td></td><td>Time = 3 hours</td><td>Cortisol / DMSO</td><td>1.45</td><td>0.227</td><td>2.390</td><td>0.0334</td></tr>
<tr><td>8 g</td><td>Saline n=3, ACTH n=4</td><td>Generalized linear mixed model, gamma distribution,</td><td>Sidak, within each group between baseline and later time points, and between treatment and control at each time point.</td><td>model_1_total=glmmTMB(Relative_HR ~ Tx *Time+ (1|ID_anita), family = Gamma(link = "log"), data=axolotl) emmeans(model_1_total, specs = trt.vs.control ~ Time|Tx, type = "response", adjust = "Tukey")

emmeans(model_1_total, specs = trt.vs.ctrl ~ Tx +Tx|Time, type = "response", adjust = "Tukey")</td><td>Tx = ACTH</td><td>1 hour / Baseline</td><td>1.43</td><td>0.0889</td><td>5.718</td><td><.0001</td></tr>
<tr><td></td><td></td><td></td><td></td><td></td><td></td><td>3 hours / Baseline</td><td>1.20</td><td>0.0747</td><td>2.929</td><td>0.0135</td></tr>
<tr><td></td><td></td><td></td><td></td><td></td><td>Time = 1 hour</td><td>ACTH / Saline</td><td>1.408</td><td>0.1244</td><td>3.870</td><td>0.0001</td></tr>
<tr><td></td><td></td><td></td><td></td><td></td><td>Time = 3 hours</td><td>ACTH / Saline</td><td>1.261</td><td>0.1115</td><td>2.627</td><td>0.0086</td></tr>
<tr><td></td><td></td><td></td><td></td><td></td><td>Time = 6 hours</td><td>ACTH / Saline</td><td>0.788</td><td>0.0697</td><td>-2.694</td><td>0.0071</td></tr>
<tr><td>8 h</td><td>Vehicle n=6, Cortisol n=6, Corticosterone n=6</td><td>Generalized linear mixed model, gamma distribution,</td><td>Sidak, within each group between baseline and later time points, and between treatment and control at each time point.</td><td>model_1_fold=glmmTMB(Fold_Change~Tx*Time+ (1|ID_anita), family = Gamma(link = "log"), data=grouped_df)

emmeans(model_1_fold, specs = trt.vs.ctrl ~ Time|Tx, type = "response", adjust = "sidak")</td><td>Tx = DMSO (Vehicle)</td><td>30 min / Baseline</td><td>1.336</td><td>0.0655</td><td>5.903</td><td><.0001</td></tr>
<tr><td></td><td></td><td></td><td></td><td></td><td></td><td>1 hour / Baseline</td><td>1.204</td><td>0.0590</td><td>3.792</td><td>0.0009</td></tr>
<tr><td></td><td></td><td></td><td></td><td></td><td>Tx = Corticosterone</td><td>30 min / Baseline</td><td>1.404</td><td>0.0688</td><td>6.916</td><td><.0001</td></tr>
<tr><td></td><td></td><td></td><td></td><td></td><td></td><td>1 hour / Baseline</td><td>1.142</td><td>0.0560</td><td>2.713</td><td>0.0393</td></tr>
<tr><td></td><td></td><td></td><td></td><td></td><td>Tx = Cortisol</td><td>30 min / Baseline</td><td>1.354</td><td>0.0664</td><td>6.189</td><td><.0001</td></tr>
<tr><td></td><td></td><td></td><td></td><td></td><td></td><td>1 hour / Baseline</td><td>1.219</td><td>0.0597</td><td>4.037</td><td>0.0003</td></tr>
<tr><td>Sup 1 a</td><td>Cortisol n=11, Corticosterone n=11</td><td>Generalized linear mixed model, gamma distribution,</td><td>Tukey, between outputs</td><td>model_1_fold=glmmTMB(Fold_Change~ Hormone*Tx*Time + (1|ID_anita), family = Gamma(link = "log"), data=grouped_df)

emmeans(model_1_fold, specs = trt.vs.ctrl ~ Hormone + Hormone|Time|Tx, type = "response", adjust = "Tukey")</td><td>Time = 1, Tx = ACTH</td><td>cortisol / corticosterone</td><td>0.244</td><td>0.0662</td><td>-5.199</td><td><.0001</td></tr>
<tr><td>1 b</td><td>Cortisol n=4, Corticosterone n=4</td><td>Generalized linear mixed model, gamma distribution</td><td>Tukey, between hormone outputs</td><td>model_1_fold=glmmTMB(Fold_Change_new~ Hormone*Tx+ (1|ID_anita), family = Gamma(link = "log"), data=grouped_df) emmeans(model_1_fold, specs = trt.vs.ctrl ~ Hormone + Hormone|Tx, type = "response", adjust = "Tukey")</td><td>Tx = ACTH</td><td>Cortisol / Corticosterone</td><td>0.565</td><td>0.0748</td><td>-4.317</td><td><.0001</td></tr>
<tr><td>1c</td><td>Cortisol n=4, Corticosterone n=4</td><td>Generalized linear mixed model, gamma distribution</td><td>Tukey, between hormone outputs</td><td>model_1_fold=glmmTMB(Fold_Change~ Hormone*Tx*Time + (1|ID_anita), family = Gamma(link = "log"), data=grouped_df) emmeans(model_1_fold, specs = trt.vs.ctrl ~ Hormone + Hormone|Time|Tx, type = "response", adjust = "Tukey")</td><td>Time = 1 hour, Tx = AVT</td><td>Cortisol / Corticosterone</td><td>0.117</td><td>0.0229</td><td>-10.960</td><td><.0001</td></tr>
<tr><td>1d</td><td>Saline n=4
Manual stress n=5,
Manual stress + MCR2 inhibitor n=4</td><td>Generalized linear mixed model, gamma distribution</td><td>Tukey, between hormone outputs</td><td>model_1_fold=glmmTMB(Fold_Change~ Hormone*Tx*Time + (1|ID_anita), family = Gamma(link = "log"), data=grouped_df) emmeans(model_1_fold, specs = trt.vs.ctrl ~ Hormone + Hormone|Time|Tx, type = "response", adjust = "Tukey") emmeans(model_1_fold, specs = pairwise ~ Tx|Time|Hormone, type = "response", adjust = "Tukey") contrastHormone_tx_relative_fold_1</td><td>Time = 5 hours, Tx = MS_MCRi</td><td>Cortisol / Corticosterone</td><td>2.959</td><td>0.529</td><td>6.065</td><td><.0001</td></tr>
<tr><td></td><td></td><td></td><td></td><td></td><td>Time = 5 hours, Tx = MS</td><td>Cortisol / Corticosterone</td><td>1.571</td><td>0.251</td><td>2.823</td><td>0.0048</td></tr>
<tr><td></td><td></td><td></td><td></td><td></td><td>Time = 5 hours, Hormone = Corticosterone</td><td>MS / MS_MCRi</td><td>1.646</td><td>0.298</td><td>2.748</td><td>0.0060</td></tr>
<tr><td></td><td></td><td></td><td></td><td></td><td>Time = 5 hours, Hormone = Cortisol</td><td>MS / MS_MCRi</td><td>0.873</td><td>0.158</td><td>-0.747</td><td>0.4550</td></tr>
</table>

1e	Saline n=4<br>Manual stress n=5,<br>Manual stress + MCR2 inhibitor n=4	Generalized linear mixed model, gamma distribution	Tukey, between hormone outputs	model_1_total=glmmTMB(Concentration_ratio ~ Tx* Hormone + (1|ID_anita), family = Gamma(link = "log"), data=baseline_df)	Tx = MS_MCRi:<br><br>Tx = MS:<br><br>Hormone = Corticosterone<br>Hormone = Cortisol	Corticosterone / Cortisol<br>Corticosterone / Cortisol<br><br>MS / MS_MCRi<br><br>MS / MS_MCRi	0.381<br>1.321<br><br>4.571<br><br>1.320	0.0539<br>0.1679<br><br>0.7376<br><br>0.2129	-6.825<br>2.187<br><br>9.420<br><br>1.723	<.0001<br>0.0288<br><br><.0001<br><br>0.1964
1f	Amputation n=10, sham n=10	Generalized linear mixed model, gamma distribution	Tukey, between hormone outputs	model_1_fold=glmmTMB(Fold_Change~ Hormone*Tx*Time + (1|ID_anita), family = Gamma(link = "log"), data=grouped_df)<br><br>emmeans(model_1_fold, specs = trt.vs.ctrl ~ Hormone + Hormone|Time|Tx, type = "response", adjust = "Tukey")	Time = 1 hour, Tx = amputation	Cortisol / Corticosterone	0.233	0.0662	-5.130	<.0001
1g	Adrenal issue taken from n=10 axolotls, each animals adrenal tissue divided into 8 sections, and paired so 2 pieces per well. n=6 for negative and positive controls and n=7 wells per treatment. Tissue measurements			model_1_total=glmmTMB(Tissue ~ Tx * Output+ (1|ID_anita:Animal), family = Gamma(link = "log"), data=axolotl)<br><br>emmeans(model_1_total, specs = pairwise ~ Output +Output|Tx, type = "response", adjust = "Tukey")	Tx = Positive control<br>Tx = ACTH<br>Tx = Epi:<br>Tx = Ach:<br>Tx = AVT:	Corticosterone / Cortisol<br>Corticosterone / Cortisol<br>Corticosterone / Cortisol<br>Corticosterone / Cortisol<br>Corticosterone / Cortisol	1.82<br>3.66<br>10.34<br>1.10<br>4.33	0.223<br>0.415<br>1.177<br>0.126<br>0.493	4.917<br>11.407<br>20.526<br>0.823<br>12.890	<.0001<br><.0001<br><.0001<br>0.4107<br><.0001
1h	Adrenal issue taken from n=10 axolotls, each animals adrenal tissue divided into 8 sections, and paired so 2 pieces per well. n=6 for negative and positive controls and n=7 wells per treatment. Media measurements			model_1_total=glmmTMB(Media ~ Tx * Output+ (1|ID_anita:Animal), family = Gamma(link = "log"), data=axolotl)<br><br>emmeans(model_1_total, specs = pairwise ~ Output +Output|Tx, type = "response", adjust = "Tukey")	Tx = Positive Control<br>Tx = ACTH<br>Tx = Adrenaline<br>Tx = Ach:<br>Tx = AVT:	Corticosterone / Cortisol<br>Corticosterone / Cortisol<br>Corticosterone / Cortisol<br>Corticosterone / Cortisol<br>Corticosterone / Cortisol	0.4964<br>1.4283<br>1.2739<br>0.0862<br>1.2751	0.0744<br>0.2010<br>0.1775<br>0.0119<br>0.1768	-4.672<br>2.533<br>1.738<br>-17.682<br>1.753	<.0001<br>0.0113<br>0.0823<br><.0001<br>0.0797

Supplementary Table 3. Recipe for Axolotl Ringer´s solution adjusted to plasma osmolarity of axolotls.

Axolotl Ringer´s Solution<br>*(Amphibian Ringer´s adjusted to Axolotl Plasma Osmolarity (208 mOsmol/ l)*<br>**Add salts as detailed and adjust to total volume of 1 liter with deionized or ultrapure water.**	
**Salt**	**[g]**
**NaCl**	5.75
**KCl**	0.13
**$CaCl_2$**	0.13
**$NaHCO_3$**	0.17
